# Adverse events associated with vismodegib: insights from a real-world pharmacovigilance study using the FAERS database

**DOI:** 10.3389/fphar.2025.1497708

**Published:** 2025-05-16

**Authors:** Bingqing Wang, Kaidi Zhao, Ningyi Xian, Landong Ren, Jiashu Liu, Chen Tu, Dewu Zhang

**Affiliations:** ^1^ Department of Dermatology, The Second Affiliated Hospital of Xi’an Jiaotong University, Xi’an, China; ^2^ Department of Dermatology, Xi’an Children’s Hospital, Xi’an, China

**Keywords:** vismodegib, adverse events, FAERS, basal cell carcinoma, disproportionality analysis

## Abstract

**Background:**

Vismodegib, an inhibitor of the Hedgehog signaling pathway, has been widely used in the treatment of advanced basal cell carcinoma. Given its critical role in managing advanced basal cell carcinoma and the relatively high rate of treatment discontinuation, it is crucial to comprehensively understand its safety profile in real-world settings.

**Methods:**

This study analyzed all adverse event reports that identified vismodegib as the primary suspected drug since 2012 in the FDA Adverse Event Reporting System database. Disproportionality analysis was conducted using four algorithms: Reporting odds ratio, proportional reporting ratio, multi-item gamma Poisson shrinker, and Bayesian confidence propagation neural network to assess the safety profile of vismodegib in clinical practice. Additionally, this study employed the Weibull distribution to model the risk of adverse events over time.

**Results:**

A total of 7,733 adverse event reports associated with vismodegib were identified from the FDA Adverse Event Reporting System database. Data mining identified positive signals for on-label adverse events, such as muscle spasms, taste alterations, alopecia, fatigue, and weight decreased. Notably, potential off-label adverse events including squamous cell carcinoma, dehydration and dysphagia were also detected. The median time to onset for these adverse events was 69 days post-drug administration, highlighting the importance of close monitoring, particularly within the initial 2 months of treatment.

**Conclusion:**

This study provides valuable insights into the real-world safety profile of vismodegib. It not only confirms the known adverse events on the label, but also suggests several potential novel adverse events, thereby supporting a more informed and rational use of vismodegib in clinical practice.

## 1 Introduction

Basal cell carcinoma (BCC) is one of the most prevalent cutaneous malignancies, particularly in the elderly, with an increasing global prevalence and growing associated healthcare burden year by year (Hu et al., 2022). Key risk factors for BCC include ultraviolet (UV) radiation and genetic mutations, with the aberrant activation of the Hedgehog (HH) signaling pathway playing a pivotal role in its pathogenesis (Peris et al., 2023; Ng and Curran, 2011).

BCC typically presents as a slow-growing tumor with limited metastatic potential. Complete surgical excision remains the first-line treatment option, with Mohs micrographic surgery regarded as the gold standard for high-risk and recurrent BCC, achieving recurrence rates of less than 6% over 5 years for both initial and recurrent BCCs. For low-risk BCC, alternative treatment modalities including destructive methods, topical therapies, and photodynamic therapy also prove effective (Kim et al., 2019; Peris et al., 2023).

However, the treatment of patients with locally advanced BCC(laBCC), metastatic BCC (mBCC), or those who are not candidates for surgery or radiotherapy remains a significant clinical challenge. In this context, vismodegib was the first FDA-approved drug for the treatment of advanced BCC (Genentech, 2023). Multiple clinical trials have demonstrated its significant efficacy for this indication (Sekulic et al., 2012; Basset-Séguin et al., 2017). In addition, studies have shown that vismodegib can alleviate the clinical manifestations of BCC with different histological subtypes in patients with Gorlin-Goltz syndrome, with an acceptable safety profile (Scotti et al., 2024a; Scotti et al., 2024b). Furthermore, a multicenter Italian study involving 52 patients with advanced BCC treated with vismodegib confirmed its effectiveness and tolerability in routine clinical practice (Mannino et al., 2023). Nevertheless, these studies are generally limited by small sample sizes, short follow-up durations, or strict inclusion and exclusion criteria, which may affect the generalizability of their findings. With the expanded post-marketing use of vismodegib, an increasing number of adverse events (AEs) have been reported, including muscle spasms, dysphagia, nausea, diarrhea (Püsküllüoğlu et al., 2024; Sekulic et al., 2017). Moreover, similar to observations with targeted therapies for melanoma (Dika et al., 2016), vismodegib has been linked to AEs involving the hair and nails (BEN ISHAI et al., 2020; Koch et al., 2024). Given the widespread use of vismodegib in routine clinical practice, a comprehensive evaluation of its safety profile in real-world populations is urgently needed and of critical importance to both clinicians and regulatory authorities.

The FDA Adverse Event Reporting System (FAERS), which aggregates voluntarily submitted AE reports from physicians, pharmacists, nurses, and consumers, plays a vital role in pharmacovigilance (Zhao et al., 2025; Zhao et al., 2024). In this study, we utilized the FAERS database to perform a disproportionality analysis to assess the safety of vismodegib in real-world settings, aiming to provide valuable safety insights to support its clinical use in the management of BCC.

## 2 Materials and methods

### 2.1 Data sources, management, and study design

**FIGURE 1:**
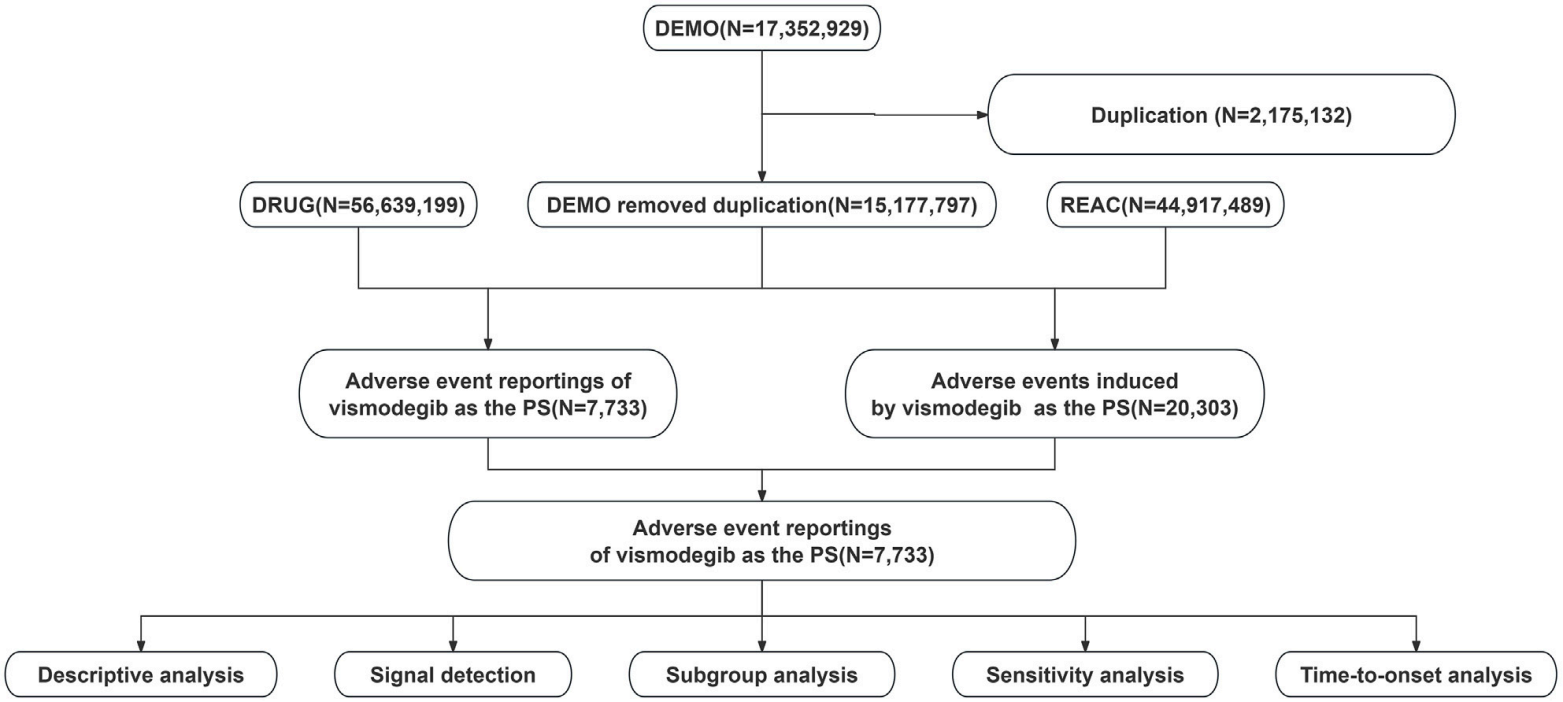
Flowchart showing the adverse event analysis process for vismodegib using the FDA Adverse Event Reporting System database.

Data for this study were obtained from the publicly available FAERS database, and AEs identifying vismodegib as the primary suspected drug were collected from the first quarter of 2012 to the first quarter of 2024. Subsequently, duplicate reports in the data were eliminated following the processing procedures recommended by FDA. For records with the same case identifiers (CASEIDs) in the demographic dataset (DEMO), the reports with the largest FDA receipt date (FDA_DT) value were retained. Similarly, for records with the same CASEID and FDA_DT, the one with the largest PRIMARYID (the unique identifier assigned to each report) value was kept. The Medical Dictionary for Regulatory Activities (MedDRA 26.1) was utilized to standardize and categorize AEs using preferred terms (PTs) and system organ classes (SOCs) in an attempt to enhance the accuracy of the data analysis that followed. Figure 1 presents a detailed flowchart of the study design.

### 2.2 Statistical analysis

The characteristics of AE reports associated with vismodegib were displayed using descriptive analysis. Disproportionality analysis was performed using two classes of four algorithms, namely, Frequentist Statistics, including reporting odds ratio (ROR) (Rothman et al., 2004), proportional reporting ratio (PRR) (Evans et al., 2001), and Bayesian Statistics, including multi-item gamma Poisson shrinker (MGPS) (Rivkees and Szarfman, 2010) and Bayesian confidence propagation neural network (BCPNN) (Ang et al., 2016). Potential AEs were defined as those meeting at least one of the positive thresholds established by these four algorithms. Supplementary Table S1 shows the detailed information on the fourfold table used for disproportionality analysis. Supplementary Table S2 displays the formulas and positive threshold intervals for each algorithm. Furthermore, the time to onset (TTO) of an AE was defined as the interval between the occurrence date of AE (EVENT-DT, recorded in the DEMO dataset) and the start date of vismodegib therapy [START-DT, recorded in the therapy (THER) dataset]. The Weibull distribution was applied to model changes in the incidence of AEs over time as well. All analyses were performed using R software version 4.2.2.

## 3 Results

### 3.1 Descriptive results

A total of 17,352,929 AE reports were obtained in the FAERS database from Q1 2012 to Q1 2024 for this study, of which 7,733 AE reports listed vismodegib as the primary suspected drug for AEs. As shown in Table 1, 56.4% of the AE reports involved female patients, while 36.7% involved male patients. The median age of the patients was 72 years [interquartile range (IQR) 61–83 years], with the maximum reports were in >65 years group (29.6%) followed by 18–65 years group (14.1%). This finding is consistent with the increased incidence of BCC with advancing age. The majority of AE reports originated from the United States, comprising 89.4% of the total. Nearly half (47.5%) of the reports were submitted by healthcare professionals. Since the approval of vismodegib for advanced BCC in 2012, AEs have been consistently reported each year, peaking in 2017 (1,440 reports, 18.6%) and remaining relatively stable in subsequent years.

**TABLE 1:** Clinical characteristics of vismodegib adverse event reports from the FAERS database (Q1 2012 - Q1 2024).

Characteristics	Case numbers	Case proportion (%)
Number of event reports	7,733	
Gender		
Male	2,841	36.7%
Female	4,362	56.4%
Miss	530	6.9%
Age(years)		
Median (IQR)	72 (61,83)	
<18	24	0.3%
18–65	1,094	14.1%
>65	2,291	29.6%
Miss	4,324	55.9%
Top 5 Reported Countries		
France	167	2.2%
United States	6,914	89.4%
Canada	81	1.0%
Germany	81	1.0%
England	58	0.8%
Reporter		
Healthcare professional	3,670	47.5%
Non-healthcare professional	3,921	50.7%
Miss	142	1.8%
Reporting year		
2012	70	0.9%
2013	199	2.6%
2014	255	3.3%
2015	429	5.5%
2016	617	8.0%
2017	1,440	18.6%
2018	829	10.7%
2019	767	9.9%
2020	710	9.2%
2021	653	8.4%
2022	725	9.4%
2023	722	9.3%
2024	317	4.1%

Abbreviation: IQR, interquartile range.

### 3.2 Signal detection of vismodegib at the SOC level

**FIGURE 2:**
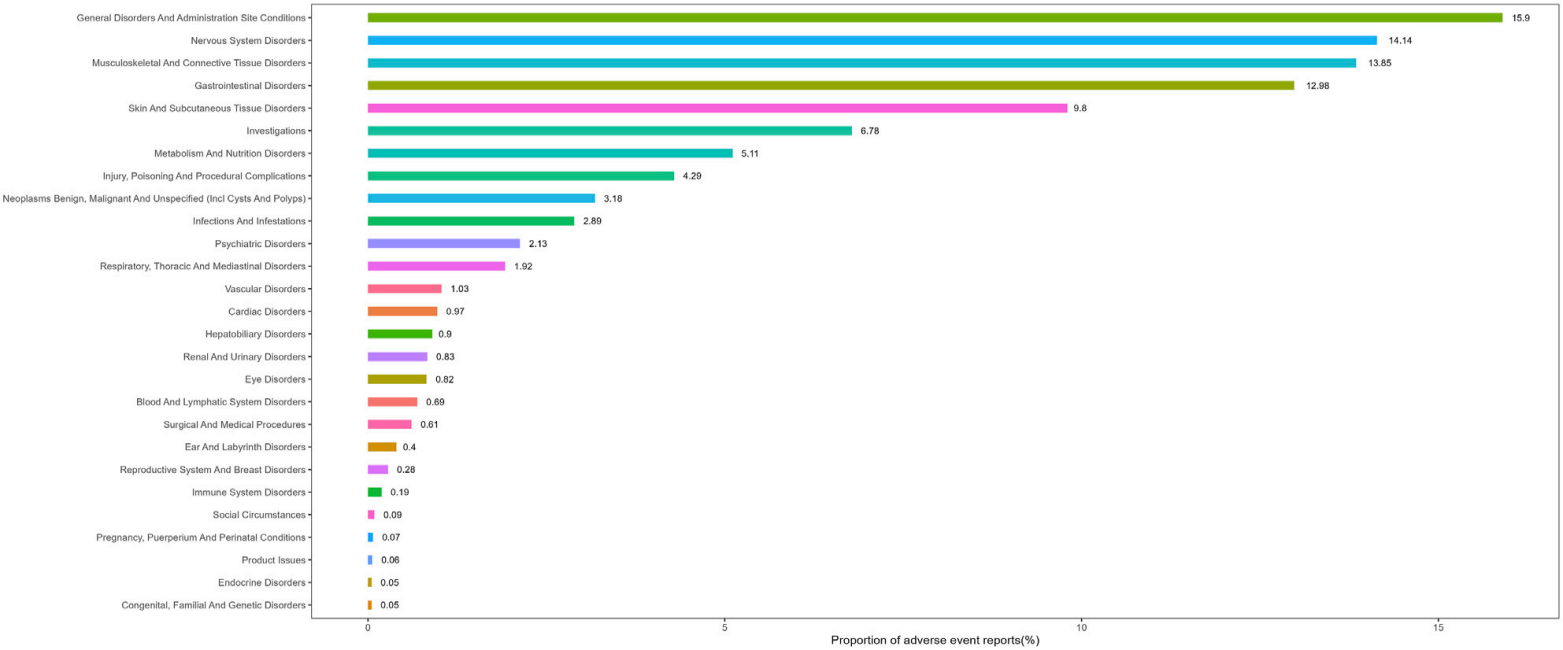
Proportion of adverse event reports by system organ class for vismodegib.

Table 2 presents the signal strength of AEs associated with vismodegib across SOC. Significantly, vismodegib-related AEs were observed in all 27 SOCs, with seven categories demonstrating positive signals: nervous system disorders, skin and subcutaneous tissue disorders, gastrointestinal disorders, musculoskeletal and connective tissue disorders, metabolism and nutrition disorders, neoplasms benign, malignant and unspecified (incl cysts and polyps) and investigations. The distribution of vismodegib-related AEs at the SOC level is illustrated in Figure 2.

**TABLE 2:** Signal strength of vismodegib AEs across system organ classes (SOC) in the FAERS database.

SOC	Case numbers	ROR (95%CI)	PRR (χ^2^)	EBGM(EBGM05)	IC(IC025)
Injury, poisoning and procedural complications	872	0.36 (0.34–0.39)	0.39 (950.13)	0.39 (0.37)	−1.37 (−3.03)
Nervous system disorders^a^	2,870	1.88 (1.8–1.95)	1.75 (1,006.5)	1.75 (1.69)	0.81 (−0.86)
Skin and subcutaneous tissue disorders^a^	1,989	1.86 (1.78–1.95)	1.78 (713.04)	1.78 (1.71)	0.83 (−0.84)
Gastrointestinal disorders^a^	2,635	1.62 (1.55–1.69)	1.54 (542.44)	1.54 (1.49)	0.62 (−1.04)
Respiratory, thoracic and mediastinal disorders	390	0.4 (0.36–0.44)	0.41 (342.03)	0.41 (0.38)	−1.28 (−2.94)
Musculoskeletal and connective tissue disorders^a^	2,811	2.87 (2.76–2.99)	2.61 (2,946.41)	2.61 (2.52)	1.38 (−0.28)
Investigations^a^	1,376	1.17 (1.11–1.24)	1.16 (32.98)	1.16 (1.11)	0.22 (−1.45)
Reproductive system and breast disorders	56	0.33 (0.25–0.42)	0.33 (77.69)	0.33 (0.26)	−1.61 (−3.27)
General disorders and administration site conditions	3,229	0.87 (0.84–0.9)	0.89 (55.28)	0.89 (0.86)	−0.17 (−1.84)
Neoplasms benign, malignant and unspecified (incl cysts and polyps)^a^	645	1.14 (1.05–1.23)	1.13 (10.51)	1.13 (1.06)	0.18 (−1.48)
Metabolism and nutrition disorders^a^	1,037	2.55 (2.39–2.71)	2.47 (924.76)	2.47 (2.34)	1.3 (−0.36)
Psychiatric disorders	433	0.38 (0.35–0.42)	0.39 (427.06)	0.39 (0.36)	−1.34 (−3.01)
Cardiac disorders	196	0.41 (0.36–0.48)	0.42 (160.23)	0.42 (0.37)	−1.25 (−2.92)
Surgical and medical procedures	124	0.45 (0.37–0.53)	0.45 (85.04)	0.45 (0.39)	−1.16 (−2.82)
Infections and infestations	586	0.52 (0.48–0.57)	0.53 (250.69)	0.53 (0.5)	−0.9 (−2.57)
Blood and lymphatic system disorders	140	0.42 (0.35–0.49)	0.42 (113.95)	0.42 (0.37)	−1.25 (−2.92)
Renal and urinary disorders	169	0.43 (0.37–0.51)	0.44 (123.29)	0.44 (0.39)	−1.19 (−2.85)
Vascular disorders	210	0.5 (0.44–0.58)	0.51 (100.83)	0.51 (0.46)	−0.97 (−2.64)
Immune system disorders	39	0.17 (0.12–0.23)	0.17 (164.41)	0.17 (0.13)	−2.58 (−4.25)
Hepatobiliary disorders	183	1.09 (0.94–1.26)	1.09 (1.24)	1.09 (0.96)	0.12 (−1.55)
Ear and labyrinth disorders	82	0.92 (0.74–1.15)	0.92 (0.53)	0.92 (0.77)	−0.12 (−1.78)
Eye disorders	167	0.42 (0.36–0.49)	0.42 (134.79)	0.42 (0.37)	−1.24 (−2.91)
Congenital, familial and genetic disorders	10	0.16 (0.09–0.31)	0.17 (42.36)	0.17 (0.1)	−2.6 (−4.26)
Pregnancy, puerperium and perinatal conditions	14	0.17 (0.1–0.29)	0.17 (55.34)	0.17 (0.11)	−2.53 (−4.19)
Social circumstances	18	0.2 (0.13–0.32)	0.2 (56.61)	0.2 (0.14)	−2.3 (−3.97)
Product issues	12	0.03 (0.02–0.06)	0.03 (324.93)	0.03 (0.02)	−4.84 (−6.5)
Endocrine disorders	10	0.19 (0.1–0.35)	0.19 (34.4)	0.19 (0.11)	−2.39 (−4.06)

Abbreviations: ROR, reporting odds ratio; PRR, proportional reporting ratio; EBGM, empirical Bayesian geometric mean; EBGM05, the lower limit of the 95% CI, of EBGM; IC, information component; IC025, the lower limit of the 95% CI, of the IC; CI, confidence interval; AEs, adverse events.

^a^ Indicate statistically significant signals in algorithm.

### 3.3 Signal detection of vismodegib at the PT level

Vismodegib-related AEs were ranked from the highest to the lowest frequency of occurrence, and the top 50 most frequent AEs were then subjected to positive signal assessment utilizing the four aforementioned algorithms. Known on-label AEs were confirmed, including muscle spasms, alopecia, ageusia, fatigue, dysgeusia, weight decreased, nausea, diarrhea, constipation, vomiting, decreased appetite, arthralgia and hepatic enzyme increased. Meanwhile, potential off-label AEs were identified such as squamous cell carcinoma (SCC), dehydration, dysphagia, myalgia, abdominal discomfort and gastrointestinal disorder. Detailed information is shown in Table 3.

**TABLE 3:** Top 50 most frequent adverse events for vismodegib at the preferred term (PT) level from FAERS.

PT	Case numbers	ROR (95%CI)	PRR (χ^2^)	EBGM(EBGM05)	IC(IC025)
Muscle spasms^a^	1,896	34.43 (32.83–36.11)	31.31 (55,016.54)	30.88 (29.68)	4.95 (3.28)
Alopecia^a^	1,293	19.41 (18.34–20.53)	18.23 (20,962.07)	18.09 (17.26)	4.18 (2.51)
Ageusia^a^	919	137.4 (128.37–147.08)	131.23 (112,157.47)	123.94 (117.08)	6.95 (5.29)
Fatigue^a^	866	3.33 (3.11–3.56)	3.23 (1,347.07)	3.22 (3.05)	1.69 (0.02)
Dysgeusia^a^	701	31.12 (28.85–33.57)	30.08 (19,466.59)	29.69 (27.87)	4.89 (3.23)
Weight decreased^a^	670	7.55 (6.99–8.16)	7.34 (3,670.8)	7.31 (6.86)	2.87 (1.2)
Nausea^a^	650	2.63 (2.43–2.85)	2.58 (635.46)	2.58 (2.41)	1.37 (−0.3)
Death^a^	644	2.23 (2.06–2.41)	2.19 (421.17)	2.19 (2.05)	1.13 (−0.54)
Decreased appetite^a^	638	8.39 (7.76–9.08)	8.16 (4,010.31)	8.14 (7.62)	3.02 (1.36)
Diarrhoea^a^	432	2.02 (1.83–2.22)	2 (216.66)	1.99 (1.84)	1 (−0.67)
Constipation^a^	353	5.11 (4.6–5.68)	5.04 (1,144.67)	5.03 (4.61)	2.33 (0.66)
Off label use	278	0.91 (0.81–1.02)	0.91 (2.51)	0.91 (0.82)	−0.14 (−1.8)
Arthralgia^a^	261	1.86 (1.65–2.1)	1.85 (102.63)	1.85 (1.67)	0.89 (−0.78)
Asthenia^a^	252	2.09 (1.84–2.36)	2.07 (140.56)	2.07 (1.87)	1.05 (−0.62)
Vomiting^a^	219	1.51 (1.32–1.72)	1.5 (36.73)	1.5 (1.34)	0.58 (−1.08)
No adverse event^a^	206	3.7 (3.23–4.25)	3.67 (401.22)	3.67 (3.27)	1.88 (0.21)
Taste disorder^a^	201	31.43 (27.33–36.15)	31.13 (5,781.77)	30.71 (27.32)	4.94 (3.27)
Myalgia^a^	190	3.61 (3.13–4.17)	3.59 (354.73)	3.58 (3.18)	1.84 (0.17)
Pain	179	0.82 (0.71–0.95)	0.82 (7.16)	0.82 (0.72)	−0.29 (−1.95)
Headache	168	0.8 (0.69–0.93)	0.8 (8.34)	0.8 (0.71)	−0.32 (−1.99)
Drug ineffective	154	0.33 (0.28–0.39)	0.33 (208.75)	0.33 (0.29)	−1.58 (−3.25)
Malaise	150	0.96 (0.82–1.13)	0.96 (0.24)	0.96 (0.84)	−0.06 (−1.72)
Rash	126	0.84 (0.7–1)	0.84 (3.87)	0.84 (0.73)	−0.25 (−1.92)
Basal cell carcinoma^a^	118	22.52 (18.77–27.01)	22.39 (2,388.26)	22.18 (19.05)	4.47 (2.8)
fall	116	1.05 (0.88–1.26)	1.05 (0.3)	1.05 (0.9)	0.07 (−1.59)
Dehydration^a^	116	2.83 (2.36–3.4)	2.82 (136.78)	2.82 (2.42)	1.5 (−0.17)
Dizziness	113	0.71 (0.59–0.85)	0.71 (13.29)	0.71 (0.61)	−0.49 (−2.16)
Abdominal pain upper^a^	108	1.62 (1.34–1.96)	1.62 (25.5)	1.62 (1.38)	0.69 (−0.97)
Squamous cell carcinoma^a^	103	34.22 (28.15–41.59)	34.05 (3,254.7)	33.55 (28.5)	5.07 (3.4)
Intentional product use issue^a^	103	3 (2.47–3.64)	2.99 (136.27)	2.99 (2.54)	1.58 (−0.09)
Insomnia	97	1.13 (0.92–1.38)	1.13 (1.42)	1.13 (0.95)	0.17 (−1.49)
Abdominal discomfort^a^	90	1.53 (1.24–1.88)	1.52 (16.27)	1.52 (1.28)	0.61 (−1.06)
Pruritus	89	0.72 (0.58–0.88)	0.72 (10)	0.72 (0.6)	−0.48 (−2.15)
Pneumonia	88	0.77 (0.63–0.95)	0.78 (5.76)	0.78 (0.65)	−0.37 (−2.03)
Pain in extremity	81	0.8 (0.64–0.99)	0.8 (4.22)	0.8 (0.66)	−0.33 (−1.99)
Dyspnoea	80	0.43 (0.35–0.54)	0.43 (60.09)	0.43 (0.36)	−1.21 (−2.87)
Abdominal pain	72	0.97 (0.77–1.23)	0.97 (0.05)	0.97 (0.8)	−0.04 (−1.71)
Dysphagia^a^	71	2.26 (1.79–2.86)	2.26 (49.85)	2.26 (1.86)	1.17 (−0.49)
Anaemia	71	1.13 (0.89–1.43)	1.13 (1.05)	1.13 (0.93)	0.18 (−1.49)
Disease progression^a^	71	1.85 (1.46–2.33)	1.85 (27.54)	1.85 (1.52)	0.88 (−0.78)
Back pain	70	0.89 (0.7–1.13)	0.89 (0.92)	0.89 (0.73)	−0.17 (−1.83)
Gastrointestinal disorder^a^	70	1.71 (1.36–2.17)	1.71 (20.75)	1.71 (1.41)	0.78 (−0.89)
Product dose omission issue	68	0.37 (0.29–0.47)	0.37 (72.16)	0.37 (0.31)	−1.42 (−3.09)
Cerebrovascular accident	63	1.28 (1–1.64)	1.28 (3.78)	1.28 (1.04)	0.35 (−1.31)
Cough	61	0.64 (0.5–0.82)	0.64 (12.46)	0.64 (0.52)	−0.64 (−2.31)
Depression	61	0.86 (0.67–1.11)	0.86 (1.32)	0.86 (0.7)	−0.21 (−1.88)
Urinary tract infection	59	1.02 (0.79–1.32)	1.02 (0.03)	1.02 (0.83)	0.03 (−1.63)
Hepatic enzyme increased^a^	58	2.75 (2.13–3.56)	2.75 (64.52)	2.75 (2.21)	1.46 (−0.21)
Pyrexia	57	0.51 (0.39–0.66)	0.51 (26.84)	0.51 (0.41)	−0.97 (−2.63)
Ill-defined disorder^a^	57	2.78 (2.14–3.6)	2.77 (64.51)	2.77 (2.23)	1.47 (−0.2)

Abbreviations: ROR, reporting odds ratio; PRR, proportional reporting ratio; EBGM, empirical Bayesian geometric mean; EBGM05, the lower limit of the 95% CI, of EBGM; IC, information component; IC025, the lower limit of the 95% CI, of the IC; CI, confidence interval; PT, preferred term.

^a^ Indicate statistically significant signals in algorithm.

### 3.4 Subgroup analysis

AEs associated with vismodegib were categorized by gender and age. AEs in each subgroup were subsequently evaluated for positive signals and the top 50 most frequent AEs in each subgroup were shown in Supplementary Tables S3–S6. The subgroup analysis revealed that insomnia and abdominal discomfort performed positive signals exclusively in male patients, while cerebrovascular accidents were unique to female patients. Among patients aged 18–65 years, the most frequent AEs with positive signal were muscle spasms, alopecia, fatigue, nausea and dysgeusia, whereas in patients over 65 years, they included muscle spasms, alopecia, fatigue, ageusia and weight decreased.

### 3.5 Sensitivity analysis

For patients with BCC, certain medications commonly used in combination with vismodegib include imiquimod, 5-fluorouracil, and mupirocin. After excluding reports that involved these concomitant medications, a total of 7,652 reports encompassing 19,997 AEs were collected. This dataset reconfirmed all previously identified potential off-label AEs, thereby verifying the robustness of the results. (Supplementary Table S7).

### 3.6 TTO and Weibull distribution analysis of AEs

**FIGURE 3:**
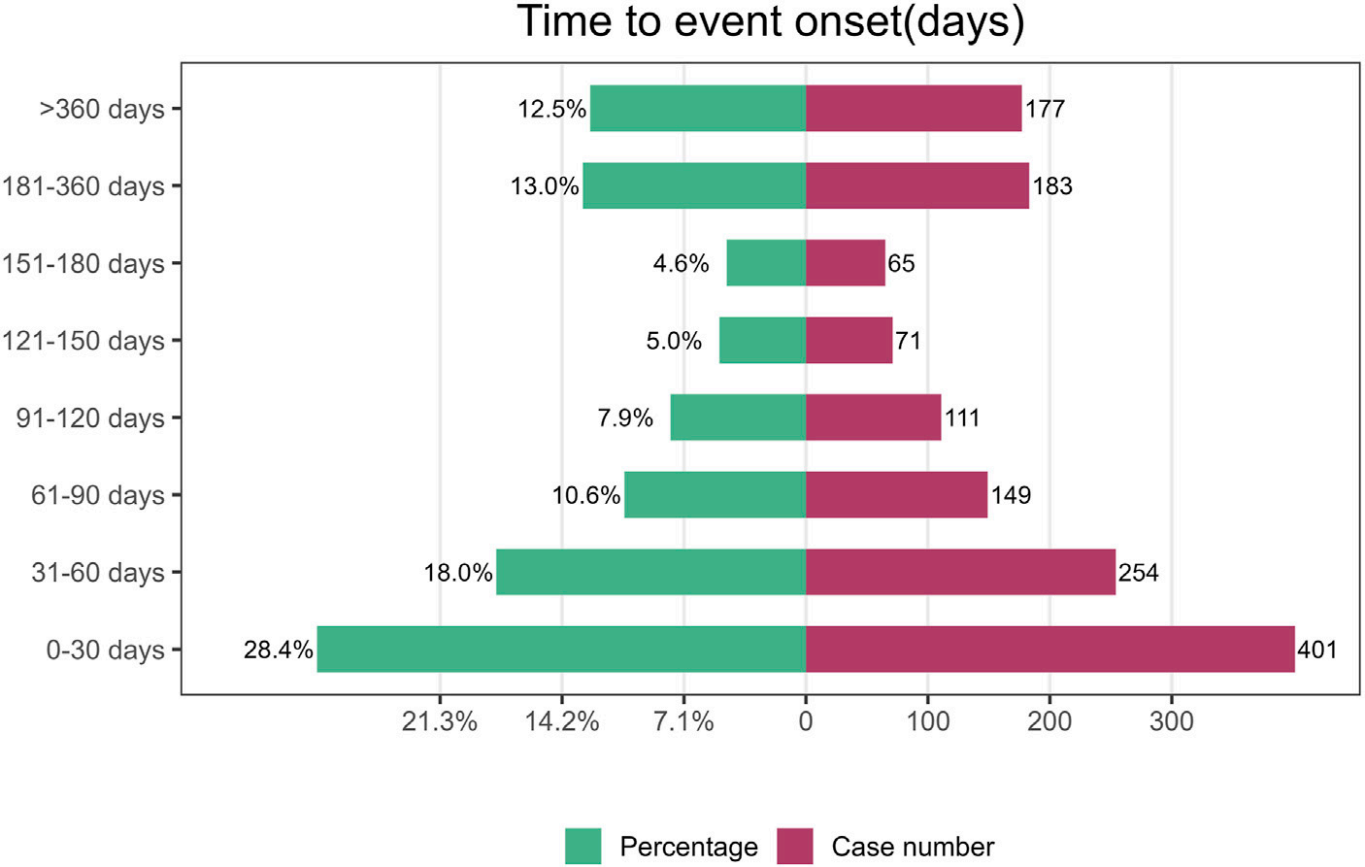
Time to onset of adverse events induced by vismodegib.

**FIGURE 4:**
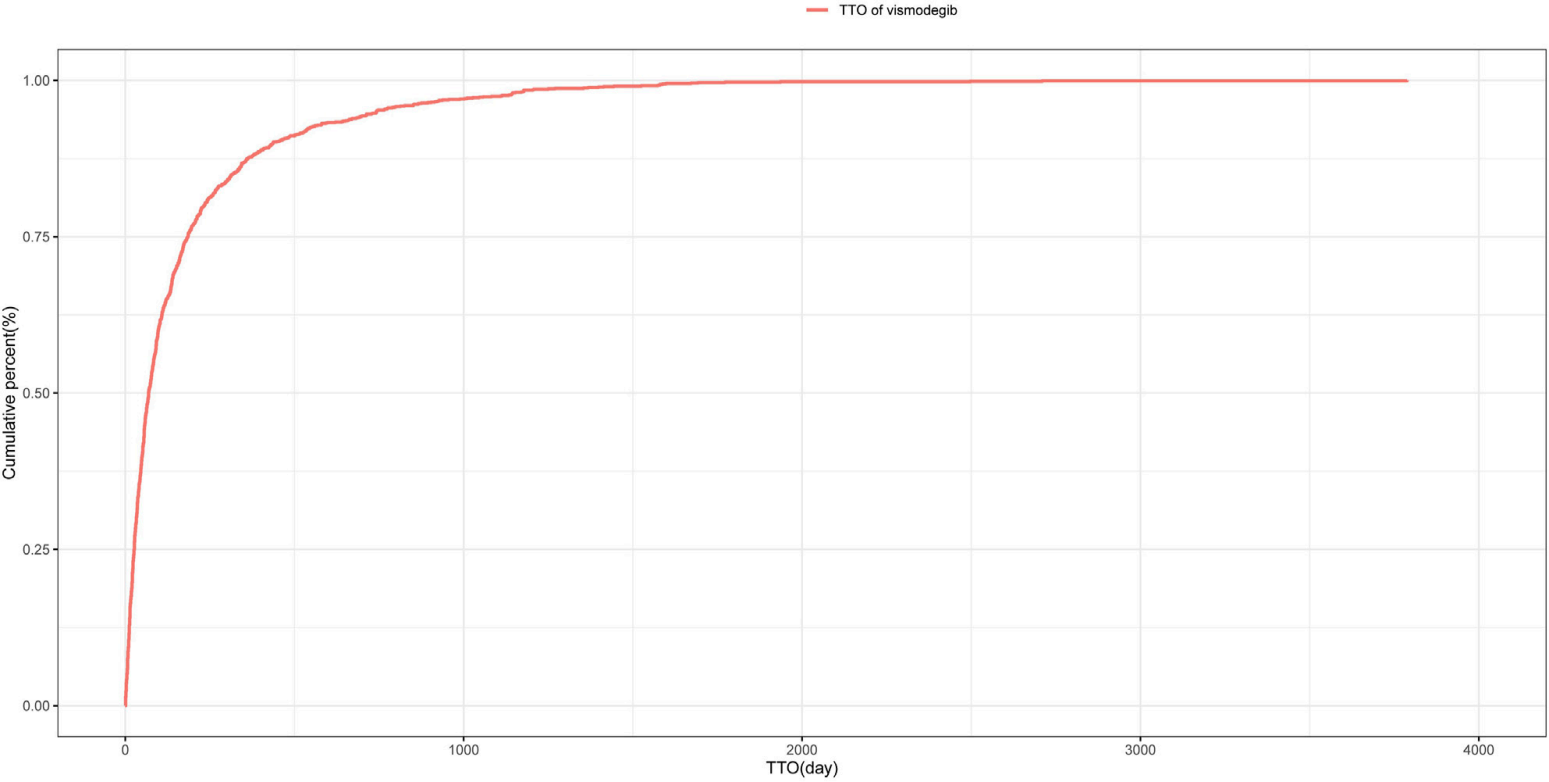
Cumulative incidence of adverse events related to vismodegib over time.

The median TTO for AEs associated with vismodegib was 69 days (IQR 27–185 days) among 1,411 reports with documented onset times that were collected from the FAERS database. As shown in Figure 3, nearly half (46.4%) of vismodegib-related AEs occurred within the first 60 days of treatment initiation, while more than a quarter (25.5%) happened after 181 days of drug administration. The cumulative incidence curve of AEs is illustrated in Figure 4. In addition, the evaluation of the Weibull Shape Parameter analysis (Table 4) showed a calculated shape parameter (β) of 0.73 with a 95% confidence interval ranging from 0.70 to 0.76, indicating a decreasing trend in the incidence of adverse effects over time, suggesting an early failure mode.

**TABLE 4:** Time to onset of vismodegib-associated adverse events and Weibull distribution analysis.

Drug	TTO (days)		Weibull distribution		
	Case reports	Median(d) (IQR)	Scale parameter: α(95% CI)	Shape parameter: β(95% CI)	Type
Vismodegib	1,411	69 (27,185)	138.2 (127.7,148.7)	0.73 (0.70,0.76)	Early failure

Abbreviation: TTO, time to onset; CI, confidence interval; IQR, interquartile range.

## 4 Discussion

This study utilized data from the FAERS database to conduct a comprehensive analysis of AEs associated with vismodegib in real-world settings. Our research confirmed several known AEs, such as muscle spasms, alopecia, fatigue, weight decreased, ageusia, dysgeusia, and nausea, which are acknowledged in the drug’s prescribing information. Additionally, the study identified potential AEs not listed in the prescribing information, including SCC, dehydration, and dysphagia. These findings underscore the necessity of drug monitoring when using vismodegib, to effectively assist clinicians in managing patient safety.

We discovered several vismodegib-related AEs consistent with those documented in the prescribing information through data analysis. Muscle spasms, alopecia, dysgeusia and ageusia were the most frequently reported AEs in clinical trials involving vismodegib, attributable to its targeted inhibition of the Hedgehog (HH) signaling pathway, which is crucial in the pathogenesis of BCC. By binding to and blocking the Smoothened, vismodegib interrupts downstream signaling within the HH cascade, thereby exerting its therapeutic effects in BCC management (Ng and Curran, 2011; Macha et al., 2013). Although this pathway remains largely quiescent in most normal adult tissues, it plays a critical role in the regulation and maintenance of certain structures, including muscle tissue, hair follicles, taste buds, and components of the reproductive system, which may explain the occurrence of these HH inhibitor (HHI)-related AEs (Macha et al., 2013; Gould et al., 2014). Understanding these AEs is essential for clinical practice, as they are generally mild to moderate in severity but tend to persist throughout treatment, potentially affecting patient tolerability and adherence.

Muscle spasms are the most prevalent AE associated with vismodegib, particularly challenging in the elderly and a major cause of treatment discontinuation. Most cases are mild to moderate, effective management of grade 1–2 muscle spasms includes non-pharmacological interventions, such as heating therapy, cryotherapy, and massage, as well as pharmacological treatments, like calcium channel blockers (e.g., amlodipine and diltiazem) (Girard et al., 2018; Siegal, 1991; Voon, 2001; Pohl et al., 2002). For severe muscle spasms (grade ≥3), treatment interruption or discontinuation may be necessary. The association between elevated creatine phosphokinase (CK) levels and HHI-induced muscle symptoms further supports the need for regular CK monitoring prior to and during therapy to guide decisions regarding dose adjustment or cessation, as indicated in the 2023 FDA label (Lear et al., 2021; Farberg et al., 2024). Alopecia induced by HHI therapy is generally reversible following cessation of therapy, but in rare circumstances can be permanent, significantly diminishing the patient’s quality of life and potentially leading to psychological distress like anxiety or depression (Gandhi et al., 2023). This highlights the importance of supportive measures, such as topical agents like minoxidil to promote hair regrowth, along with cosmetic approaches like wigs or hairpieces to enhance self-esteem and psychological wellbeing. Dysgeusia and ageusia, involving altered or lost taste perception, can negatively impact oral intake, leading to decreased appetite, weight decreased, and subsequent nutritional deficiencies, further complicating the overall clinical condition of the patient. Management may require a multidisciplinary approach, including nutritional counseling, tailored dietary modifications, and the use of flavor enhancers to improve food intake. Recent research also indicates that supplementation with brain-derived neurotrophic factor (BDNF) or its downstream mediators may help reduce taste impairment and dysfunction when administered either before or during vismodegib therapy, providing a potential strategy to address these taste disorders (Mohan and Chang, 2015; Jacobsen et al., 2017; Vukmanovic Nosrat et al., 2022).

Additionally, gastrointestinal AEs, including nausea, diarrhea, constipation, and vomiting, as well as systemic effects such as fatigue, decreased appetite, weight decreased, and hepatic enzymes increased, have been frequently observed in clinical trials involving vismodegib. Severe gastrointestinal symptoms may result in electrolyte imbalances and further weight decreased, compromising treatment adherence and therapeutic effectiveness. Pharmacologic interventions, such as antiemetics for nausea and antidiarrheals for diarrhea, can help alleviate these symptoms. Although routine monitoring of hepatic enzyme levels is not explicitly required on the drug label, increased levels may indicate liver impairment, warranting regular monitoring of hepatic enzymes to detect early hepatotoxicity and prevent further progression (Farberg et al., 2024). These AEs, which are not fatal but can compromise patient adherence and drug efficacy, should be paid much attention to ensure that patients insist and benefit optimally from the treatment.

Of note, this study also identified several potential off-label AEs associated with vismodegib that are not mentioned in the prescribing information, underscoring their clinical significance. Among these, the development of SCC is particularly concerning, the underlying mechanism may involve the inhibition of the HH signaling pathway could activate alternative pathways, such as the RAS/MAPK pathway, thereby promoting tumor growth and enhancing metastatic potential (Zhao et al., 2015). This presents a significant risk, especially considering that secondary SCC has been observed in multiple clinical trials and case reports involving BCC patients treated with vismodegib, particularly in elderly patients and those with lesions located on sun-exposed sites (Sekulic et al., 2017; Mohan et al., 2016; Basset-Séguin et al., 2017). A retrospective cohort study in 2017 did not find a statistically significant association between vismodegib use and the subsequent risk of SCC development (Bhutani et al., 2017). In contrast, a case-control study in 2016 showed that the incidence of secondary SCC was significantly higher in BCC patients exposed to vismodegib compared to those who were not (Mohan et al., 2016). Given the potential rapid progression and poor prognosis of secondary SCC, especially in patients with BCC treated with vismodegib, regular skin examinations and close dermatological monitoring are essential for early detection and timely intervention.

Dehydration has also emerged as a potential concern associated with vismodegib, although the exact mechanisms by which the drug may lead to this condition are not fully understood. Dehydration may be linked to other AEs, such as ageusia, dysgeusia, and decreased appetite, and can further aggravate symptoms like muscle spasms and fatigue, creating a cycle of worsening clinical manifestations. Given that patients with BCC are generally older, dehydration in this population can exacerbate existing comorbidities and elevate the risk of adverse health outcomes (Li et al., 2023b). The current gold standard for diagnosing dehydration relies on biochemical tests, such as serum osmolality and, occasionally, the blood urea nitrogen (BUN) to creatinine ratio. However, these tests are often impractical or inaccessible for patients with BCC, making timely monitoring of dehydration challenging and thereby increasing the risk of severe complications, such as electrolyte imbalances, renal dysfunction, cardiovascular issues, and even death (Hooper et al., 2015; Miller, 2015). Therefore, prompt recognition and proactive management of dehydration are essential to preventing these potentially severe outcomes and ensuring patient safety.

Additionally, dysphagia, though not widely reported in previous literature, may emerge as a concern during vismodegib therapy. Its occurrence could be associated with other AEs, such as dysgeusia, or may be a consequence of the underlying malignancy itself. Dysphagia poses significant risks by severely compromising nutritional intake, which can lead to malnutrition, dehydration, and aspiration. It may also result in potentially life-threatening complications, such as aspiration pneumonia or airway obstruction, particularly in high-risk patient populations. Given its potential to substantially worsen clinical outcomes, regular clinical swallow assessments should be considered, including instrumental evaluations when necessary (Mulheren et al., 2022; Okuni et al., 2021; McCarty and Chao, 2021; Rommel and Hamdy, 2016). Management of dysphagia is inherently multidisciplinary, typically involving collaboration among clinicians, speech and language pathologists, and dietitians. Furthermore, emerging evidence suggests that enhancing taste perception through targeted interventions may improve swallowing function, offering a potential strategy for addressing this AE (Mulheren et al., 2022).

The subgroup analysis highlights the need for targeted monitoring of specific AEs in different gender populations. In male patients, particular attention should be given to insomnia and abdominal discomfort, while in female patients, cerebrovascular accidents warrant increased vigilance. Notably, insomnia has been linked to numerous comorbidities, including cardiovascular diseases and cancer (Julie et al., 2020), further highlighting the need for vigilant monitoring during vismodegib therapy. Cerebrovascular accidents represent a significant clinical concern due to their potential severity. Interestingly, the role of HH signaling pathway activation in the pathogenesis of cerebrovascular diseases remains controversial and warrants further investigation (Zhang et al., 2023; Li et al., 2023a). Additionally, individuals aged over 65 years should be particularly alert to the occurrence of ageusia, which may impact their nutritional status and overall health.

The sensitivity analysis, which excluded concomitant medications, reaffirmed several AEs previously documented in the prescribing information, including muscle spasms, alopecia, dysgeusia, ageusia, gastrointestinal symptoms, and systemic effects. While most of these AEs were mild to moderate, they often persisted, potentially affecting patient tolerance and adherence to therapy. In more severe cases, they may lead to a deterioration in patient’s overall clinical condition, making early identification and intervention essential. More concerning are the potential off-label AEs, including SCC, dehydration, and dysphagia, which also demonstrated positive signals in the sensitivity analysis. These effects can significantly impair the quality of life, exacerbate existing health conditions, and, in severe cases, lead to life-threatening complications, particularly given their lack of specific manifestations in the early stages. These findings underscore the critical need for vigilant monitoring and prompt management of these AEs even those not currently included in the drug’s prescribing information. Healthcare providers should remain alert to these potential risks, employing regular monitoring and proactive management strategies to minimize their impact.

This study analyzed TTO of AEs related to vismodegib, using the Weibull distribution to model their time distribution, which highlighted the critical importance of close monitoring during the first 2 months of treatment, particularly within the first month. Additionally, ongoing surveillance throughout the entire therapy course is crucial. Nearly all patients treated with vismodegib experienced at least one treatment-related AE, significantly contributing to drug discontinuation, with most AEs-related discontinuation occurring early in the treatment (Sekulic et al., 2012). Consequently, educating patients about potential AEs and ensuring early detection and appropriate management are vital for improving adherence.

Given the high incidence of AEs related to vismodegib, flexible dosing strategies may help reduce the frequency of severe AEs and enhance both the safety and efficacy of the medication. The FDA currently recommends a daily dose of 150 mg until disease progression or unacceptable toxicity occurs, with dose interruptions allowed for up to 8 weeks if severe AEs are observed. However, maintaining long-term treatment at a tolerable and effective dose remains challenging. Clinical trials and *post hoc* analyses have suggested that dose reductions or intermittent dosing can preserve therapeutic efficacy while reducing the occurrence of severe AEs, thereby lowering discontinuation rates (Dréno et al., 2017; Chanu et al., 2021; Wong et al., 2020). These findings support the potential benefit of flexible dosing to manage AEs and improve patient adherence, although further studies are needed to determine the optimal dosing regimen. Additionally, the preoperative use of vismodegib has shown efficacy in downstaging tumors, particularly in cases of laBCC located in functionally sensitive areas, allowing discontinuation once an optimal response is achieved without the need for prolonged use (Bertrand et al., 2021; Kwon et al., 2016). Therefore, early recognition of AEs, comprehensive patient education, and effective management strategies are essential to enhance patient safety and optimize treatment outcomes.

Notably, both vismodegib and sonidegib are HH signaling pathway inhibitors approved for laBCC, yet they exhibit clinically significant pharmacokinetic differences that influence their clinical use (Genentech, 2023). Vismodegib demonstrates concentration-dependent plasma protein binding and limited tissue distribution, coupled with a shorter half-life (4–12 days) that enables rapid steady-state achievement and quicker resolution of AEs upon discontinuation (Graham et al., 2012; Genentech, 2023). In contrast, sonidegib displays non-concentration-dependent plasma protein binding and extensive tissue distribution, along with a prolonged half-life (28–30 days) that results in delayed steady-state attainment and prolonged AE persistence post-treatment (Dummer et al., 2020; Zollinger et al., 2014). Therefore, when choosing medication for laBCC, healthcare providers should carefully consider these differences to optimize outcomes and minimize the impact of AEs on patients.

Beyond intrinsic drug safety profiles, drug interactions represent a critical consideration for therapeutic efficacy and patient safety. Vismodegib has PH-dependent solubility and is metabolized by certain cytochrome P (CYP) 450 enzymes *in vitro* (Wong et al., 2009), indicating potential interactions with drugs affecting these metabolic pathways, so caution is needed when it is used with antacids or systemic antifungal drugs. The European Summary of Product Characteristics (SPC) specifically states that concomitant use with strong CYP inducers such as rifampicin, carbamazepine, and phenytoin should be avoided, as these may substantially reduce vismodegib plasma concentrations and compromise treatment efficacy (ROCHE, 2021). Moreover, *in vitro* studies show that vismodegib is an inhibitor of the breast cancer resistance protein (BCRP) transporter (Zhang et al., 2009), potentially increasing systemic exposure to BCRP substrate drugs like statins and topotecan. This interaction is particularly relevant for elderly BCC patients receiving statins for hyperlipidemia or cancer patients requiring concomitant topotecan therapy (Chaturvedi et al., 2016; Roche, 2021). When co-administering vismodegib with these drugs, healthcare providers should be cautious, and may need to adjust the dosage. Thus, further research, including large-scale clinical trials and mechanistic studies, is necessary to fully understand these interactions and safeguard patient safety.

While this study provides valuable insights, several limitations should be acknowledged. The data derived from the FAERS database may be affected by inaccuracies or incomplete information due to its reliance on voluntary submissions from consumers, physicians or pharmacists. This approach can introduce reporting bias, as some reports may lack essential details, such as comorbidities, concomitant medications, dosages, and duration. Furthermore, since most of the reports included in this analysis originated from the United States, the generalizability of the findings to other regions may be limited. Future studies should aim to include more comprehensive data from diverse countries. Additionally, this study was not designed to determine precise incidence rates or establish definitive causal relationships between vismodegib exposure and AEs, necessitating further epidemiologic research and well-structured prospective studies to clarify these associations. Despite these limitations, utilizing the FAERS database for pharmacovigilance offers the significant advantage of accessing a large, real-world dataset, which can help in the early detection of drug safety signals and trends across a broad population.

## 5 Conclusion

This study conducted a comprehensive analysis of AEs associated with vismodegib using data from the FAERS database. It confirmed known AEs and identified potential off-label AEs such as SCC, dehydration and dysphagia. Given the potential for AEs to interrupt medication use, this study emphasizes the necessity of monitoring adverse reactions during vismodegib treatment. Additionally, this research provides guidance on safe medication use for clinicians administering vismodegib, thereby enhancing patient safety and compliance with the treatment regimen.

## Data Availability

The original contributions presented in the study are included in the article/Supplementary Material, further inquiries can be directed to the corresponding authors.
