# Supplementary material for: Adverse events associated with vismodegib: insights from a real-world pharmacovigilance study using the FAERS database
Source: Front Pharmacol. 2025 May 16;16:1497708. doi: 10.3389/fphar.2025.1497708 (PMC12122506; doi:10.3389/fphar.2025.1497708)
Supplement: Supplementary file 1 [file Table1.docx]

Supplementary Material

Supplementary Tables

Supplementary Table 1:

Two-by-two contingency table for disproportionality analyses.

|  | Target AEs | Other AEs | Total |
| --- | --- | --- | --- |
| Vismodegib | a | b | a+b |
| Other drugs | c | d | c+d |
| Total | a+c | b+d | a+b+c+d |

Abbreviation: AEs, adverse events; a, number of reports containing both the target drug and target adverse drug reaction; b, number of reports containing other adverse drug reaction of the target drug; c, number of reports containing the target adverse drug reaction of other drugs; d, number of reports containing other drugs and other adverse drug reactions.

Supplementary Table 2:

Four major algorithms used for signal detection.

| Algorithms | Equation | Criteria |
| --- | --- | --- |
| ROR | ROR=ad/b/c | lower limit of 95% CI>1, N≥3 |
|  | 95%CI=e^ln(ROR)±1.96(1/a+1/b+1/c+1/d)^0.5^ |  |
| PRR | PRR=a(c+d)/c/(a+b) | PRR≥2, χ^2^≥4, N≥3 |
|  | χ^2^=[(ad-bc)^2](a+b+c+d)/[(a+b)(c+d)(a+c)(b+d)] |  |
| BCPNN | IC=log_2_a(a+b+c+d)(a+c)(a+b) | IC025>0 |
|  | 95%CI= E(IC) ± 2V(IC)^0.5 |  |
| MGPS | EBGM=a(a+b+c+d)/(a+c)/(a+b) | EBGM05>2 |
|  | 95%CI=e^ln(EBGM)±1.96(1/a+1/b+1/c+1/d)^0.5^ |  |

Abbreviation: a, number of reports containing both the target drug and target adverse drug reaction; b, number of reports containing other adverse drug reaction of the target drug; c, number of reports containing the target adverse drug reaction of other drugs; d, number of reports containing other drugs and other adverse drug reactions. 95%CI, 95% confidence interval; N, the number of reports; χ2, chi-squared; IC, information component; IC025, the lower limit of 95% CI of the IC; E(IC), the IC expectations; V(IC), the variance of IC; EBGM, empirical Bayesian geometric mean; EBGM05, the lower limit of 95% CI of EBGM.

Supplementary Table 3:

Top 50 most frequent adverse events for vismodegib at the preferred term (PT) level in males from FAERS data

| PT | Case numbers | ROR(95%CI) | PRR(χ^2^) | EBGM(EBGM05) | IC(IC025) |
| --- | --- | --- | --- | --- | --- |
| Muscle spasms* | 1,142 | 45.64 ( 42.89 - 48.56 ) | 41.08 ( 43436.06 ) | 39.88 ( 37.87 ) | 5.32 ( 3.65 ) |
| Alopecia* | 613 | 60.55 ( 55.72 - 65.79 ) | 57.29 ( 32540.82 ) | 54.97 ( 51.28 ) | 5.78 ( 4.11 ) |
| Ageusia* | 585 | 140.25 ( 128.55 - 153.01 ) | 132.97 ( 69716.93 ) | 121.03 ( 112.52 ) | 6.92 ( 5.25 ) |
| Fatigue* | 467 | 3.45 ( 3.14 - 3.79 ) | 3.35 ( 776.6 ) | 3.34 ( 3.09 ) | 1.74 ( 0.07 ) |
| Weight decreased* | 399 | 7.14 ( 6.46 - 7.9 ) | 6.92 ( 2022.13 ) | 6.89 ( 6.34 ) | 2.79 ( 1.12 ) |
| Decreased appetite* | 383 | 8.05 ( 7.27 - 8.92 ) | 7.81 ( 2270.61 ) | 7.77 ( 7.13 ) | 2.96 ( 1.29 ) |
| Dysgeusia* | 383 | 34 ( 30.67 - 37.69 ) | 32.87 ( 11562.87 ) | 32.1 ( 29.45 ) | 5 ( 3.34 ) |
| Death* | 373 | 1.71 ( 1.54 - 1.9 ) | 1.69 ( 106.88 ) | 1.69 ( 1.55 ) | 0.76 ( -0.91 ) |
| Nausea* | 302 | 2.98 ( 2.66 - 3.34 ) | 2.92 ( 385.12 ) | 2.92 ( 2.65 ) | 1.55 ( -0.12 ) |
| Diarrhoea* | 245 | 2.11 ( 1.86 - 2.4 ) | 2.09 ( 140.37 ) | 2.09 ( 1.88 ) | 1.06 ( -0.6 ) |
| Constipation* | 222 | 5.91 ( 5.18 - 6.76 ) | 5.82 ( 884.79 ) | 5.8 ( 5.19 ) | 2.54 ( 0.87 ) |
| Asthenia* | 141 | 1.96 ( 1.66 - 2.31 ) | 1.94 ( 64.96 ) | 1.94 ( 1.69 ) | 0.96 ( -0.71 ) |
| Arthralgia* | 138 | 2.22 ( 1.88 - 2.63 ) | 2.21 ( 91.74 ) | 2.21 ( 1.92 ) | 1.14 ( -0.52 ) |
| Off label use | 129 | 0.78 ( 0.66 - 0.93 ) | 0.78 ( 7.72 ) | 0.78 ( 0.68 ) | -0.35 ( -2.02 ) |
| Taste disorder* | 123 | 33.35 ( 27.86 - 39.92 ) | 33 ( 3725.6 ) | 32.23 ( 27.72 ) | 5.01 ( 3.34 ) |
| Myalgia* | 115 | 4.1 ( 3.41 - 4.93 ) | 4.07 ( 266.39 ) | 4.06 ( 3.48 ) | 2.02 ( 0.36 ) |
| No adverse event* | 101 | 4.63 ( 3.8 - 5.63 ) | 4.59 ( 283.65 ) | 4.58 ( 3.89 ) | 2.2 ( 0.53 ) |
| Vomiting* | 98 | 1.48 ( 1.22 - 1.81 ) | 1.48 ( 15.28 ) | 1.48 ( 1.25 ) | 0.56 ( -1.1 ) |
| Pain | 96 | 0.9 ( 0.74 - 1.11 ) | 0.9 ( 0.97 ) | 0.91 ( 0.76 ) | -0.14 ( -1.81 ) |
| Drug ineffective | 87 | 0.37 ( 0.3 - 0.45 ) | 0.37 ( 94.69 ) | 0.37 ( 0.31 ) | -1.43 ( -3.1 ) |
| Dizziness | 78 | 0.98 ( 0.78 - 1.22 ) | 0.98 ( 0.03 ) | 0.98 ( 0.81 ) | -0.03 ( -1.7 ) |
| Malaise | 77 | 1.06 ( 0.85 - 1.33 ) | 1.06 ( 0.3 ) | 1.06 ( 0.88 ) | 0.09 ( -1.58 ) |
| Headache | 71 | 0.83 ( 0.66 - 1.05 ) | 0.83 ( 2.41 ) | 0.83 ( 0.68 ) | -0.26 ( -1.93 ) |
| Fall | 70 | 1.2 ( 0.95 - 1.52 ) | 1.2 ( 2.38 ) | 1.2 ( 0.99 ) | 0.26 ( -1.4 ) |
| Dehydration* | 70 | 2.79 ( 2.2 - 3.53 ) | 2.78 ( 79.53 ) | 2.77 ( 2.28 ) | 1.47 ( -0.2 ) |
| Basal cell carcinoma* | 68 | 18.78 ( 14.77 - 23.88 ) | 18.67 ( 1122.1 ) | 18.43 ( 15.08 ) | 4.2 ( 2.54 ) |
| Squamous cell carcinoma* | 67 | 27.74 ( 21.76 - 35.35 ) | 27.58 ( 1681.81 ) | 27.04 ( 22.07 ) | 4.76 ( 3.09 ) |
| Rash | 63 | 0.8 ( 0.63 - 1.03 ) | 0.8 ( 3.09 ) | 0.8 ( 0.65 ) | -0.32 ( -1.98 ) |
| Insomnia* | 61 | 1.35 ( 1.05 - 1.73 ) | 1.34 ( 5.41 ) | 1.34 ( 1.09 ) | 0.43 ( -1.24 ) |
| Abdominal pain upper* | 59 | 1.95 ( 1.51 - 2.52 ) | 1.95 ( 27.18 ) | 1.94 ( 1.57 ) | 0.96 ( -0.71 ) |
| Pneumonia | 56 | 0.75 ( 0.58 - 0.98 ) | 0.75 ( 4.59 ) | 0.75 ( 0.6 ) | -0.41 ( -2.08 ) |
| Abdominal discomfort* | 55 | 2 ( 1.53 - 2.61 ) | 1.99 ( 27.31 ) | 1.99 ( 1.6 ) | 1 ( -0.67 ) |
| Intentional product use issue* | 51 | 2.86 ( 2.17 - 3.77 ) | 2.85 ( 61.36 ) | 2.85 ( 2.26 ) | 1.51 ( -0.16 ) |
| Product dose omission issue | 48 | 0.49 ( 0.37 - 0.65 ) | 0.49 ( 25.81 ) | 0.49 ( 0.39 ) | -1.03 ( -2.7 ) |
| Pruritus | 47 | 0.8 ( 0.6 - 1.06 ) | 0.8 ( 2.37 ) | 0.8 ( 0.63 ) | -0.32 ( -1.99 ) |
| Pain in extremity | 47 | 1.05 ( 0.79 - 1.4 ) | 1.05 ( 0.1 ) | 1.05 ( 0.82 ) | 0.07 ( -1.6 ) |
| Disease progression* | 41 | 1.65 ( 1.22 - 2.25 ) | 1.65 ( 10.5 ) | 1.65 ( 1.28 ) | 0.72 ( -0.94 ) |
| Anaemia | 40 | 1.04 ( 0.76 - 1.42 ) | 1.04 ( 0.06 ) | 1.04 ( 0.8 ) | 0.06 ( -1.61 ) |
| Dyspnoea | 39 | 0.37 ( 0.27 - 0.51 ) | 0.38 ( 40.47 ) | 0.38 ( 0.29 ) | -1.41 ( -3.07 ) |
| Back pain | 36 | 0.93 ( 0.67 - 1.3 ) | 0.93 ( 0.17 ) | 0.93 ( 0.71 ) | -0.1 ( -1.76 ) |
| Gastrointestinal disorder* | 35 | 1.91 ( 1.37 - 2.66 ) | 1.91 ( 15.09 ) | 1.91 ( 1.44 ) | 0.93 ( -0.74 ) |
| Cough | 35 | 0.71 ( 0.51 - 0.98 ) | 0.71 ( 4.27 ) | 0.71 ( 0.54 ) | -0.5 ( -2.17 ) |
| Dysphagia* | 35 | 1.85 ( 1.33 - 2.58 ) | 1.84 ( 13.55 ) | 1.84 ( 1.4 ) | 0.88 ( -0.78 ) |
| Abdominal pain | 34 | 0.9 ( 0.65 - 1.27 ) | 0.9 ( 0.34 ) | 0.9 ( 0.68 ) | -0.14 ( -1.81 ) |
| Pyrexia | 34 | 0.48 ( 0.34 - 0.67 ) | 0.48 ( 18.9 ) | 0.48 ( 0.36 ) | -1.05 ( -2.71 ) |
| Cerebrovascular accident | 34 | 1.01 ( 0.72 - 1.42 ) | 1.01 ( 0.01 ) | 1.01 ( 0.77 ) | 0.02 ( -1.65 ) |
| Myocardial infarction | 33 | 0.84 ( 0.59 - 1.18 ) | 0.84 ( 1.06 ) | 0.84 ( 0.63 ) | -0.26 ( -1.92 ) |
| Neoplasm malignant* | 32 | 2.34 ( 1.65 - 3.3 ) | 2.33 ( 24.32 ) | 2.33 ( 1.74 ) | 1.22 ( -0.45 ) |
| Hypogeusia* | 32 | 81.38 ( 56.93 - 116.34 ) | 81.15 ( 2388.36 ) | 76.56 ( 56.78 ) | 6.26 ( 4.59 ) |
| Anosmia* | 31 | 18.16 ( 12.73 - 25.89 ) | 18.11 ( 494.44 ) | 17.88 ( 13.29 ) | 4.16 ( 2.49 ) |

Abbreviation: Asterisks (*) indicate statistically significant signals in algorithm; ROR, reporting odds ratio; PRR, proportional reporting ratio; EBGM, empirical Bayesian geometric mean; EBGM05, the lower limit of the 95% CI of EBGM; IC, information component; IC025, the lower limit of the 95% CI of the IC; CI, confidence interval; PT,preferred term; AEs, adverse events.

Supplementary Table 4:

Top 50 most frequent adverse events for vismodegib at the PT level in females from FAERS data

| PT | Case numbers | ROR(95%CI) | PRR(χ^2^) | EBGM(EBGM05) | IC(IC025) |
| --- | --- | --- | --- | --- | --- |
| Muscle spasms* | 631 | 24.59 ( 22.66 - 26.68 ) | 22.75 ( 13073 ) | 22.6 ( 21.11 ) | 4.5 ( 2.83 ) |
| Alopecia* | 595 | 16.54 ( 15.21 - 17.98 ) | 15.4 ( 8011.21 ) | 15.33 ( 14.29 ) | 3.94 ( 2.27 ) |
| Fatigue* | 373 | 3.38 ( 3.04 - 3.75 ) | 3.27 ( 595.11 ) | 3.27 ( 2.99 ) | 1.71 ( 0.04 ) |
| Nausea* | 321 | 2.78 ( 2.48 - 3.11 ) | 2.71 ( 350.27 ) | 2.71 ( 2.46 ) | 1.44 ( -0.23 ) |
| Ageusia* | 295 | 113.11 ( 100.5 - 127.3 ) | 109.03 ( 30520.89 ) | 105.38 ( 95.46 ) | 6.72 ( 5.05 ) |
| Dysgeusia* | 276 | 28.24 ( 25.03 - 31.85 ) | 27.31 ( 6943.71 ) | 27.08 ( 24.48 ) | 4.76 ( 3.09 ) |
| Weight decreased* | 239 | 7.12 ( 6.26 - 8.1 ) | 6.94 ( 1218.35 ) | 6.93 ( 6.22 ) | 2.79 ( 1.13 ) |
| Decreased appetite* | 239 | 8.24 ( 7.24 - 9.37 ) | 8.03 ( 1471.75 ) | 8.01 ( 7.19 ) | 3 ( 1.34 ) |
| Death* | 229 | 2.82 ( 2.48 - 3.22 ) | 2.77 ( 261.94 ) | 2.77 ( 2.48 ) | 1.47 ( -0.2 ) |
| Diarrhoea* | 168 | 1.88 ( 1.62 - 2.19 ) | 1.86 ( 68.04 ) | 1.86 ( 1.64 ) | 0.9 ( -0.77 ) |
| Off label use | 125 | 1.15 ( 0.96 - 1.37 ) | 1.15 ( 2.4 ) | 1.15 ( 0.99 ) | 0.2 ( -1.47 ) |
| Constipation* | 124 | 4.52 ( 3.78 - 5.39 ) | 4.46 ( 333.79 ) | 4.46 ( 3.84 ) | 2.16 ( 0.49 ) |
| Arthralgia* | 116 | 1.8 ( 1.5 - 2.16 ) | 1.79 ( 40.54 ) | 1.79 ( 1.53 ) | 0.84 ( -0.83 ) |
| Vomiting* | 108 | 1.65 ( 1.37 - 2 ) | 1.65 ( 27.56 ) | 1.65 ( 1.4 ) | 0.72 ( -0.95 ) |
| Asthenia* | 104 | 2.14 ( 1.77 - 2.6 ) | 2.13 ( 62.54 ) | 2.13 ( 1.81 ) | 1.09 ( -0.58 ) |
| Headache | 91 | 0.91 ( 0.74 - 1.12 ) | 0.91 ( 0.84 ) | 0.91 ( 0.76 ) | -0.14 ( -1.8 ) |
| Pain | 78 | 0.8 ( 0.64 - 1 ) | 0.8 ( 3.92 ) | 0.8 ( 0.66 ) | -0.32 ( -1.99 ) |
| Malaise | 70 | 0.98 ( 0.78 - 1.24 ) | 0.98 ( 0.02 ) | 0.98 ( 0.81 ) | -0.03 ( -1.69 ) |
| No adverse event* | 70 | 3.88 ( 3.07 - 4.91 ) | 3.85 ( 148.09 ) | 3.85 ( 3.16 ) | 1.95 ( 0.28 ) |
| Taste disorder* | 68 | 26.69 ( 21 - 33.92 ) | 26.48 ( 1653.37 ) | 26.26 ( 21.49 ) | 4.71 ( 3.05 ) |
| Myalgia* | 63 | 2.85 ( 2.22 - 3.65 ) | 2.83 ( 74.75 ) | 2.83 ( 2.3 ) | 1.5 ( -0.17 ) |
| Rash | 57 | 0.91 ( 0.7 - 1.18 ) | 0.91 ( 0.49 ) | 0.91 ( 0.73 ) | -0.13 ( -1.8 ) |
| Drug ineffective | 48 | 0.26 ( 0.2 - 0.35 ) | 0.27 ( 97.42 ) | 0.27 ( 0.21 ) | -1.89 ( -3.56 ) |
| Abdominal pain upper* | 47 | 1.54 ( 1.15 - 2.05 ) | 1.53 ( 8.71 ) | 1.53 ( 1.2 ) | 0.62 ( -1.05 ) |
| Intentional product use issue* | 47 | 4.45 ( 3.34 - 5.92 ) | 4.43 ( 124.61 ) | 4.42 ( 3.48 ) | 2.14 ( 0.48 ) |
| Fall | 45 | 0.93 ( 0.69 - 1.24 ) | 0.93 ( 0.25 ) | 0.93 ( 0.73 ) | -0.11 ( -1.77 ) |
| Dehydration* | 42 | 2.61 ( 1.93 - 3.54 ) | 2.6 ( 41.47 ) | 2.6 ( 2.02 ) | 1.38 ( -0.29 ) |
| Dyspnoea | 39 | 0.51 ( 0.37 - 0.7 ) | 0.51 ( 18.55 ) | 0.51 ( 0.39 ) | -0.97 ( -2.64 ) |
| Pruritus | 38 | 0.69 ( 0.5 - 0.95 ) | 0.69 ( 5.34 ) | 0.69 ( 0.53 ) | -0.54 ( -2.2 ) |
| Basal cell carcinoma* | 38 | 21.18 ( 15.38 - 29.16 ) | 21.08 ( 722.29 ) | 20.95 ( 16.03 ) | 4.39 ( 2.72 ) |
| Abdominal pain | 34 | 1.07 ( 0.76 - 1.49 ) | 1.06 ( 0.13 ) | 1.06 ( 0.8 ) | 0.09 ( -1.58 ) |
| Urinary tract infection | 33 | 1.12 ( 0.8 - 1.58 ) | 1.12 ( 0.45 ) | 1.12 ( 0.84 ) | 0.17 ( -1.5 ) |
| Dizziness | 33 | 0.47 ( 0.34 - 0.67 ) | 0.48 ( 19.26 ) | 0.48 ( 0.36 ) | -1.07 ( -2.74 ) |
| Pain in extremity | 33 | 0.68 ( 0.48 - 0.95 ) | 0.68 ( 5.09 ) | 0.68 ( 0.51 ) | -0.56 ( -2.23 ) |
| Insomnia | 32 | 0.88 ( 0.62 - 1.25 ) | 0.88 ( 0.51 ) | 0.88 ( 0.66 ) | -0.18 ( -1.85 ) |
| Depression | 32 | 1.05 ( 0.74 - 1.49 ) | 1.05 ( 0.08 ) | 1.05 ( 0.79 ) | 0.07 ( -1.6 ) |
| Abdominal discomfort | 31 | 1.18 ( 0.83 - 1.68 ) | 1.18 ( 0.87 ) | 1.18 ( 0.88 ) | 0.24 ( -1.43 ) |
| Pneumonia | 31 | 0.74 ( 0.52 - 1.05 ) | 0.74 ( 2.84 ) | 0.74 ( 0.55 ) | -0.43 ( -2.1 ) |
| Gastrointestinal disorder* | 31 | 1.82 ( 1.28 - 2.59 ) | 1.81 ( 11.34 ) | 1.81 ( 1.35 ) | 0.86 ( -0.81 ) |
| Madarosis* | 30 | 10.92 ( 7.62 - 15.64 ) | 10.88 ( 268.36 ) | 10.85 ( 8.03 ) | 3.44 ( 1.77 ) |
| Dysphagia* | 29 | 2.44 ( 1.69 - 3.51 ) | 2.43 ( 24.46 ) | 2.43 ( 1.79 ) | 1.28 ( -0.39 ) |
| Hepatic enzyme increased* | 28 | 3.11 ( 2.15 - 4.51 ) | 3.1 ( 39.94 ) | 3.1 ( 2.27 ) | 1.63 ( -0.03 ) |
| Cerebrovascular accident* | 28 | 1.68 ( 1.16 - 2.44 ) | 1.68 ( 7.74 ) | 1.68 ( 1.23 ) | 0.75 ( -0.92 ) |
| Cough | 26 | 0.63 ( 0.43 - 0.92 ) | 0.63 ( 5.66 ) | 0.63 ( 0.46 ) | -0.67 ( -2.33 ) |
| Squamous cell carcinoma* | 25 | 31.65 ( 21.33 - 46.97 ) | 31.56 ( 732.45 ) | 31.25 ( 22.47 ) | 4.97 ( 3.3 ) |
| Back pain | 25 | 0.71 ( 0.48 - 1.05 ) | 0.71 ( 3.06 ) | 0.71 ( 0.51 ) | -0.5 ( -2.17 ) |
| Anaemia | 25 | 1.1 ( 0.75 - 1.63 ) | 1.1 ( 0.24 ) | 1.1 ( 0.79 ) | 0.14 ( -1.52 ) |
| Disease progression* | 23 | 1.96 ( 1.3 - 2.95 ) | 1.96 ( 10.76 ) | 1.96 ( 1.39 ) | 0.97 ( -0.7 ) |
| Anxiety | 23 | 0.57 ( 0.38 - 0.87 ) | 0.58 ( 7.22 ) | 0.58 ( 0.41 ) | -0.8 ( -2.46 ) |
| Ill-defined disorder* | 23 | 3.99 ( 2.65 - 6.01 ) | 3.98 ( 51.29 ) | 3.98 ( 2.82 ) | 1.99 ( 0.33 ) |

Abbreviation: Asterisks (*) indicate statistically significant signals in algorithm; ROR, reporting odds ratio; PRR, proportional reporting ratio; EBGM, empirical Bayesian geometric mean; EBGM05, the lower limit of the 95% CI of EBGM; IC, information component; IC025, the lower limit of the 95% CI of the IC; CI, confidence interval; PT,preferred term; AEs, adverse events.

Supplementary Table 5:

Top 50 most frequent adverse events for vismodegib at the PT level in patients aged 18 to 65 from FAERS data

| PT | Case numbers | ROR(95%CI) | PRR(χ^2^) | EBGM(EBGM05) | IC(IC025) |
| --- | --- | --- | --- | --- | --- |
| Muscle spasms* | 350 | 34.4 ( 30.8 - 38.43 ) | 31.11 ( 10164.69 ) | 30.91 ( 28.18 ) | 4.95 ( 3.28 ) |
| Alopecia* | 191 | 14.79 ( 12.78 - 17.12 ) | 14.05 ( 2317.49 ) | 14.01 ( 12.4 ) | 3.81 ( 2.14 ) |
| Fatigue* | 153 | 3.16 ( 2.69 - 3.72 ) | 3.07 ( 216.36 ) | 3.07 ( 2.68 ) | 1.62 ( -0.05 ) |
| Nausea* | 122 | 2.44 ( 2.04 - 2.93 ) | 2.4 ( 100.53 ) | 2.39 ( 2.06 ) | 1.26 ( -0.41 ) |
| Dysgeusia* | 108 | 26.21 ( 21.63 - 31.76 ) | 25.45 ( 2525.96 ) | 25.32 ( 21.56 ) | 4.66 ( 3 ) |
| Ageusia* | 104 | 99.75 ( 81.92 - 121.47 ) | 96.86 ( 9670.93 ) | 94.93 ( 80.5 ) | 6.57 ( 4.9 ) |
| Decreased appetite* | 86 | 7.41 ( 5.98 - 9.18 ) | 7.25 ( 464.39 ) | 7.24 ( 6.05 ) | 2.86 ( 1.19 ) |
| Weight decreased* | 76 | 5.36 ( 4.27 - 6.72 ) | 5.26 ( 263.25 ) | 5.26 ( 4.35 ) | 2.39 ( 0.73 ) |
| Diarrhoea* | 70 | 1.97 ( 1.55 - 2.49 ) | 1.95 ( 32.59 ) | 1.95 ( 1.6 ) | 0.96 ( -0.71 ) |
| Death* | 55 | 2.12 ( 1.63 - 2.77 ) | 2.1 ( 32.09 ) | 2.1 ( 1.68 ) | 1.07 ( -0.59 ) |
| Vomiting* | 52 | 1.81 ( 1.38 - 2.39 ) | 1.8 ( 18.74 ) | 1.8 ( 1.43 ) | 0.85 ( -0.82 ) |
| Off label use | 50 | 1.09 ( 0.83 - 1.44 ) | 1.09 ( 0.38 ) | 1.09 ( 0.86 ) | 0.12 ( -1.54 ) |
| Arthralgia* | 49 | 1.74 ( 1.31 - 2.31 ) | 1.73 ( 15.26 ) | 1.73 ( 1.37 ) | 0.79 ( -0.87 ) |
| Headache | 46 | 1.01 ( 0.75 - 1.35 ) | 1.01 ( 0 ) | 1.01 ( 0.79 ) | 0.01 ( -1.66 ) |
| Myalgia* | 43 | 4.23 ( 3.13 - 5.72 ) | 4.19 ( 104.83 ) | 4.19 ( 3.26 ) | 2.07 ( 0.4 ) |
| Pain | 42 | 1.09 ( 0.81 - 1.48 ) | 1.09 ( 0.32 ) | 1.09 ( 0.85 ) | 0.12 ( -1.54 ) |
| Constipation* | 41 | 4.28 ( 3.14 - 5.82 ) | 4.24 ( 101.66 ) | 4.24 ( 3.27 ) | 2.08 ( 0.42 ) |
| Asthenia* | 31 | 1.56 ( 1.09 - 2.22 ) | 1.55 ( 6.16 ) | 1.55 ( 1.16 ) | 0.64 ( -1.03 ) |
| Neutrophil count decreased* | 28 | 10.85 ( 7.48 - 15.74 ) | 10.77 ( 247.82 ) | 10.75 ( 7.87 ) | 3.43 ( 1.76 ) |
| Taste disorder* | 28 | 31.56 ( 21.73 - 45.83 ) | 31.31 ( 816.49 ) | 31.11 ( 22.77 ) | 4.96 ( 3.29 ) |
| Abdominal pain* | 25 | 1.54 ( 1.04 - 2.28 ) | 1.54 ( 4.71 ) | 1.54 ( 1.11 ) | 0.62 ( -1.05 ) |
| Disease progression* | 25 | 4.19 ( 2.83 - 6.22 ) | 4.17 ( 60.35 ) | 4.17 ( 3 ) | 2.06 ( 0.39 ) |
| Dehydration* | 24 | 3.86 ( 2.59 - 5.77 ) | 3.84 ( 50.57 ) | 3.84 ( 2.75 ) | 1.94 ( 0.28 ) |
| Anaemia* | 24 | 2.55 ( 1.7 - 3.81 ) | 2.54 ( 22.4 ) | 2.54 ( 1.81 ) | 1.34 ( -0.32 ) |
| Back pain | 22 | 1.49 ( 0.98 - 2.27 ) | 1.49 ( 3.55 ) | 1.49 ( 1.05 ) | 0.57 ( -1.09 ) |
| Pneumonia | 21 | 1.24 ( 0.8 - 1.9 ) | 1.23 ( 0.94 ) | 1.23 ( 0.86 ) | 0.3 ( -1.36 ) |
| Abdominal pain upper* | 20 | 1.56 ( 1.01 - 2.42 ) | 1.56 ( 4.02 ) | 1.56 ( 1.08 ) | 0.64 ( -1.03 ) |
| Insomnia | 20 | 1.21 ( 0.78 - 1.87 ) | 1.21 ( 0.71 ) | 1.21 ( 0.83 ) | 0.27 ( -1.4 ) |
| Malaise | 19 | 0.72 ( 0.46 - 1.14 ) | 0.73 ( 1.99 ) | 0.73 ( 0.5 ) | -0.46 ( -2.13 ) |
| White blood cell count decreased* | 18 | 2.62 ( 1.65 - 4.17 ) | 2.61 ( 17.98 ) | 2.61 ( 1.77 ) | 1.39 ( -0.28 ) |
| Drug ineffective | 17 | 0.26 ( 0.16 - 0.43 ) | 0.27 ( 34.6 ) | 0.27 ( 0.18 ) | -1.9 ( -3.57 ) |
| No adverse event* | 17 | 4.02 ( 2.5 - 6.48 ) | 4.01 ( 38.42 ) | 4.01 ( 2.69 ) | 2 ( 0.34 ) |
| Hyponatraemia* | 16 | 7.76 ( 4.75 - 12.69 ) | 7.73 ( 93.65 ) | 7.72 ( 5.12 ) | 2.95 ( 1.28 ) |
| Lymphocyte count decreased* | 16 | 9.95 ( 6.09 - 16.27 ) | 9.91 ( 127.99 ) | 9.89 ( 6.56 ) | 3.31 ( 1.64 ) |
| Intentional product use issue* | 16 | 3.15 ( 1.93 - 5.15 ) | 3.14 ( 23.33 ) | 3.14 ( 2.08 ) | 1.65 ( -0.02 ) |
| Dizziness | 15 | 0.52 ( 0.31 - 0.86 ) | 0.52 ( 6.65 ) | 0.52 ( 0.34 ) | -0.94 ( -2.61 ) |
| Dyspnoea | 15 | 0.47 ( 0.28 - 0.78 ) | 0.47 ( 8.93 ) | 0.47 ( 0.31 ) | -1.08 ( -2.75 ) |
| Rash | 15 | 0.58 ( 0.35 - 0.96 ) | 0.58 ( 4.64 ) | 0.58 ( 0.38 ) | -0.79 ( -2.46 ) |
| Squamous cell carcinoma* | 14 | 37.01 ( 21.85 - 62.69 ) | 36.87 ( 484.85 ) | 36.59 ( 23.55 ) | 5.19 ( 3.53 ) |
| Pruritus | 14 | 0.59 ( 0.35 - 1 ) | 0.59 ( 3.99 ) | 0.59 ( 0.38 ) | -0.76 ( -2.43 ) |
| Embolism* | 14 | 39.6 ( 23.38 - 67.09 ) | 39.45 ( 520.39 ) | 39.13 ( 25.18 ) | 5.29 ( 3.62 ) |
| Product dose omission issue | 13 | 0.42 ( 0.24 - 0.72 ) | 0.42 ( 10.36 ) | 0.42 ( 0.27 ) | -1.24 ( -2.91 ) |
| Basal cell carcinoma* | 13 | 16.47 ( 9.55 - 28.43 ) | 16.42 ( 187.61 ) | 16.36 ( 10.37 ) | 4.03 ( 2.37 ) |
| Hypophosphataemia* | 13 | 28.83 ( 16.7 - 49.79 ) | 28.73 ( 345.88 ) | 28.56 ( 18.08 ) | 4.84 ( 3.17 ) |
| Platelet count decreased* | 12 | 2.33 ( 1.32 - 4.1 ) | 2.32 ( 9.05 ) | 2.32 ( 1.44 ) | 1.22 ( -0.45 ) |
| Aspartate aminotransferase increased* | 12 | 4.09 ( 2.32 - 7.22 ) | 4.08 ( 27.94 ) | 4.08 ( 2.54 ) | 2.03 ( 0.36 ) |
| Ill-defined disorder* | 12 | 6.52 ( 3.7 - 11.49 ) | 6.5 ( 55.8 ) | 6.49 ( 4.04 ) | 2.7 ( 1.03 ) |
| Depression | 12 | 0.81 ( 0.46 - 1.42 ) | 0.81 ( 0.54 ) | 0.81 ( 0.5 ) | -0.31 ( -1.97 ) |
| Seizure | 12 | 1.31 ( 0.75 - 2.32 ) | 1.31 ( 0.9 ) | 1.31 ( 0.82 ) | 0.39 ( -1.27 ) |
| Pain in extremity | 12 | 0.61 ( 0.35 - 1.08 ) | 0.61 ( 2.97 ) | 0.61 ( 0.38 ) | -0.71 ( -2.38 ) |

Abbreviation: Asterisks (*) indicate statistically significant signals in algorithm; ROR, reporting odds ratio; PRR, proportional reporting ratio; EBGM, empirical Bayesian geometric mean; EBGM05, the lower limit of the 95% CI of EBGM; IC, information component; IC025, the lower limit of the 95% CI of the IC; CI, confidence interval; PT, preferred term.

Supplementary Table 6:

Top 50 most frequent adverse events for vismodegib at the PT level in patients aged over 65 from FAERS data

| PT | Case numbers | ROR(95%CI) | PRR(χ^2^) | EBGM(EBGM05) | IC(IC025) |
| --- | --- | --- | --- | --- | --- |
| Muscle spasms* | 472 | 25.93 ( 23.6 - 28.5 ) | 24.1 ( 10319.73 ) | 23.74 ( 21.94 ) | 4.57 ( 2.9 ) |
| Death* | 335 | 2.54 ( 2.27 - 2.83 ) | 2.46 ( 295.63 ) | 2.46 ( 2.24 ) | 1.3 ( -0.37 ) |
| Alopecia* | 324 | 21.57 ( 19.28 - 24.14 ) | 20.53 ( 5954.97 ) | 20.27 ( 18.45 ) | 4.34 ( 2.68 ) |
| Fatigue* | 243 | 2.92 ( 2.57 - 3.32 ) | 2.85 ( 294.64 ) | 2.84 ( 2.55 ) | 1.51 ( -0.16 ) |
| Ageusia* | 241 | 81.42 ( 71.36 - 92.89 ) | 78.39 ( 17526.19 ) | 74.63 ( 66.83 ) | 6.22 ( 4.55 ) |
| Weight decreased* | 232 | 6.99 ( 6.13 - 7.97 ) | 6.77 ( 1142.49 ) | 6.75 ( 6.04 ) | 2.75 ( 1.09 ) |
| Decreased appetite* | 226 | 6.37 ( 5.58 - 7.28 ) | 6.18 ( 983.35 ) | 6.16 ( 5.51 ) | 2.62 ( 0.96 ) |
| Dysgeusia* | 207 | 25 ( 21.74 - 28.74 ) | 24.22 ( 4543.29 ) | 23.86 ( 21.23 ) | 4.58 ( 2.91 ) |
| Nausea* | 169 | 2.31 ( 1.98 - 2.69 ) | 2.27 ( 121.98 ) | 2.27 ( 2 ) | 1.18 ( -0.48 ) |
| Diarrhoea* | 138 | 1.6 ( 1.35 - 1.9 ) | 1.59 ( 30.61 ) | 1.59 ( 1.38 ) | 0.67 ( -1 ) |
| Constipation* | 126 | 4.41 ( 3.7 - 5.27 ) | 4.35 ( 325.32 ) | 4.34 ( 3.74 ) | 2.12 ( 0.45 ) |
| Asthenia* | 105 | 1.9 ( 1.56 - 2.3 ) | 1.88 ( 43.62 ) | 1.88 ( 1.6 ) | 0.91 ( -0.76 ) |
| Arthralgia* | 74 | 1.78 ( 1.41 - 2.23 ) | 1.77 ( 24.79 ) | 1.77 ( 1.46 ) | 0.82 ( -0.85 ) |
| Vomiting* | 65 | 1.48 ( 1.16 - 1.89 ) | 1.48 ( 10.13 ) | 1.48 ( 1.2 ) | 0.56 ( -1.1 ) |
| Fall | 65 | 0.99 ( 0.78 - 1.27 ) | 0.99 ( 0.01 ) | 0.99 ( 0.81 ) | -0.01 ( -1.68 ) |
| Taste disorder* | 58 | 20.73 ( 15.98 - 26.89 ) | 20.55 ( 1064.94 ) | 20.29 ( 16.32 ) | 4.34 ( 2.68 ) |
| Myalgia* | 57 | 3.07 ( 2.36 - 3.99 ) | 3.05 ( 78.71 ) | 3.05 ( 2.45 ) | 1.61 ( -0.06 ) |
| Off label use | 48 | 0.51 ( 0.38 - 0.67 ) | 0.51 ( 22.91 ) | 0.51 ( 0.4 ) | -0.97 ( -2.64 ) |
| Malaise | 47 | 0.89 ( 0.67 - 1.19 ) | 0.89 ( 0.61 ) | 0.89 ( 0.7 ) | -0.16 ( -1.83 ) |
| Squamous cell carcinoma* | 45 | 29.55 ( 21.98 - 39.73 ) | 29.35 ( 1209.41 ) | 28.82 ( 22.5 ) | 4.85 ( 3.18 ) |
| Pain | 45 | 0.9 ( 0.67 - 1.21 ) | 0.9 ( 0.49 ) | 0.9 ( 0.71 ) | -0.15 ( -1.82 ) |
| Dehydration* | 42 | 2.04 ( 1.51 - 2.77 ) | 2.04 ( 22.2 ) | 2.04 ( 1.58 ) | 1.03 ( -0.64 ) |
| Dizziness | 39 | 0.66 ( 0.48 - 0.91 ) | 0.66 ( 6.67 ) | 0.66 ( 0.51 ) | -0.59 ( -2.26 ) |
| Rash | 38 | 0.89 ( 0.64 - 1.22 ) | 0.89 ( 0.55 ) | 0.89 ( 0.68 ) | -0.17 ( -1.84 ) |
| Basal cell carcinoma* | 35 | 14.45 ( 10.35 - 20.18 ) | 14.38 ( 431.84 ) | 14.26 ( 10.78 ) | 3.83 ( 2.17 ) |
| Pneumonia | 35 | 0.63 ( 0.45 - 0.88 ) | 0.63 ( 7.52 ) | 0.63 ( 0.48 ) | -0.66 ( -2.33 ) |
| Pyrexia | 34 | 0.94 ( 0.67 - 1.32 ) | 0.94 ( 0.12 ) | 0.94 ( 0.71 ) | -0.08 ( -1.75 ) |
| Headache | 34 | 0.7 ( 0.5 - 0.98 ) | 0.7 ( 4.43 ) | 0.7 ( 0.53 ) | -0.52 ( -2.18 ) |
| Pain in extremity | 33 | 0.9 ( 0.64 - 1.27 ) | 0.9 ( 0.35 ) | 0.9 ( 0.68 ) | -0.15 ( -1.81 ) |
| Drug ineffective | 33 | 0.35 ( 0.25 - 0.5 ) | 0.36 ( 38.51 ) | 0.36 ( 0.27 ) | -1.48 ( -3.15 ) |
| Urinary tract infection | 32 | 1.2 ( 0.85 - 1.69 ) | 1.2 ( 1.02 ) | 1.2 ( 0.89 ) | 0.26 ( -1.41 ) |
| Abdominal pain upper | 30 | 1.43 ( 1 - 2.05 ) | 1.43 ( 3.88 ) | 1.43 ( 1.06 ) | 0.51 ( -1.15 ) |
| Anaemia | 29 | 0.87 ( 0.6 - 1.25 ) | 0.87 ( 0.6 ) | 0.87 ( 0.64 ) | -0.21 ( -1.87 ) |
| Cerebrovascular accident | 29 | 1.25 ( 0.87 - 1.8 ) | 1.25 ( 1.42 ) | 1.25 ( 0.92 ) | 0.32 ( -1.35 ) |
| Cough | 28 | 0.79 ( 0.55 - 1.15 ) | 0.79 ( 1.55 ) | 0.79 ( 0.58 ) | -0.34 ( -2 ) |
| Pruritus | 28 | 0.74 ( 0.51 - 1.08 ) | 0.74 ( 2.47 ) | 0.74 ( 0.55 ) | -0.43 ( -2.09 ) |
| Insomnia | 28 | 1.17 ( 0.81 - 1.69 ) | 1.17 ( 0.68 ) | 1.17 ( 0.86 ) | 0.22 ( -1.44 ) |
| Hepatic enzyme increased* | 26 | 5.27 ( 3.58 - 7.75 ) | 5.25 ( 89.22 ) | 5.24 ( 3.79 ) | 2.39 ( 0.72 ) |
| Dysphagia* | 25 | 1.98 ( 1.34 - 2.94 ) | 1.98 ( 12.09 ) | 1.98 ( 1.42 ) | 0.98 ( -0.68 ) |
| Abdominal pain | 24 | 1.11 ( 0.74 - 1.65 ) | 1.1 ( 0.24 ) | 1.1 ( 0.79 ) | 0.14 ( -1.52 ) |
| Neoplasm malignant* | 24 | 3.62 ( 2.42 - 5.41 ) | 3.61 ( 45.28 ) | 3.61 ( 2.58 ) | 1.85 ( 0.18 ) |
| No adverse event* | 24 | 4.43 ( 2.97 - 6.62 ) | 4.42 ( 63.34 ) | 4.41 ( 3.15 ) | 2.14 ( 0.47 ) |
| Myocardial infarction | 23 | 1.19 ( 0.79 - 1.79 ) | 1.19 ( 0.7 ) | 1.19 ( 0.84 ) | 0.25 ( -1.42 ) |
| Gastrointestinal disorder* | 22 | 2.1 ( 1.38 - 3.19 ) | 2.09 ( 12.54 ) | 2.09 ( 1.47 ) | 1.06 ( -0.6 ) |
| Dyspnoea | 22 | 0.28 ( 0.18 - 0.43 ) | 0.28 ( 40.57 ) | 0.28 ( 0.2 ) | -1.82 ( -3.49 ) |
| Therapy cessation* | 22 | 3.89 ( 2.56 - 5.92 ) | 3.88 ( 47.04 ) | 3.88 ( 2.73 ) | 1.95 ( 0.29 ) |
| Product dose omission issue | 21 | 0.47 ( 0.31 - 0.72 ) | 0.47 ( 12.4 ) | 0.47 ( 0.33 ) | -1.08 ( -2.75 ) |
| Disease progression | 21 | 1.49 ( 0.97 - 2.29 ) | 1.49 ( 3.38 ) | 1.49 ( 1.04 ) | 0.57 ( -1.09 ) |
| Covid-19 | 21 | 0.88 ( 0.57 - 1.34 ) | 0.88 ( 0.37 ) | 0.88 ( 0.61 ) | -0.19 ( -1.86 ) |
| General physical health deterioration | 19 | 1.1 ( 0.7 - 1.73 ) | 1.1 ( 0.18 ) | 1.1 ( 0.76 ) | 0.14 ( -1.53 ) |

Abbreviation: Asterisks (*) indicate statistically significant signals in algorithm; ROR, reporting odds ratio; PRR, proportional reporting ratio; EBGM, empirical Bayesian geometric mean; EBGM05, the lower limit of the 95% CI of EBGM; IC, information component; IC025, the lower limit of the 95% CI of the IC; CI, confidence interval; PT, preferred term.

Supplementary Table 7:

Top 50 most frequent adverse events for vismodegib excluding common medication co-usage at the PT level from FAERS data

| PT | Case numbers | ROR(95%CI) | PRR(χ^2^) | EBGM(EBGM05) | IC(IC025) |
| --- | --- | --- | --- | --- | --- |
| Muscle spasms* | 1,873 | 34.54 ( 32.92 - 36.23 ) | 31.4 ( 54521.91 ) | 30.98 ( 29.76 ) | 4.95 ( 3.29 ) |
| Alopecia* | 1,276 | 19.44 ( 18.37 - 20.58 ) | 18.27 ( 20731.47 ) | 18.13 ( 17.28 ) | 4.18 ( 2.51 ) |
| Ageusia* | 911 | 138.26 ( 129.14 - 148.04 ) | 132.01 ( 111910.73 ) | 124.74 ( 117.81 ) | 6.96 ( 5.3 ) |
| Fatigue* | 853 | 3.33 ( 3.11 - 3.56 ) | 3.23 ( 1327 ) | 3.22 ( 3.04 ) | 1.69 ( 0.02 ) |
| Dysgeusia* | 687 | 30.95 ( 28.67 - 33.42 ) | 29.92 ( 18976.16 ) | 29.54 ( 27.71 ) | 4.88 ( 3.22 ) |
| Weight decreased* | 663 | 7.59 ( 7.02 - 8.2 ) | 7.37 ( 3654.88 ) | 7.35 ( 6.89 ) | 2.88 ( 1.21 ) |
| Death* | 638 | 2.24 ( 2.07 - 2.42 ) | 2.2 ( 423.8 ) | 2.2 ( 2.06 ) | 1.14 ( -0.53 ) |
| Nausea* | 635 | 2.61 ( 2.41 - 2.82 ) | 2.56 ( 609.37 ) | 2.56 ( 2.39 ) | 1.35 ( -0.31 ) |
| Decreased appetite* | 624 | 8.33 ( 7.69 - 9.03 ) | 8.1 ( 3887.25 ) | 8.08 ( 7.56 ) | 3.01 ( 1.35 ) |
| Diarrhoea* | 424 | 2.01 ( 1.83 - 2.21 ) | 1.99 ( 210.41 ) | 1.99 ( 1.83 ) | 0.99 ( -0.67 ) |
| Constipation* | 346 | 5.09 ( 4.57 - 5.66 ) | 5.02 ( 1113.85 ) | 5.01 ( 4.58 ) | 2.32 ( 0.66 ) |
| Off label use | 272 | 0.9 ( 0.8 - 1.02 ) | 0.9 ( 2.81 ) | 0.9 ( 0.82 ) | -0.15 ( -1.81 ) |
| Arthralgia* | 254 | 1.84 ( 1.62 - 2.08 ) | 1.83 ( 95.9 ) | 1.83 ( 1.65 ) | 0.87 ( -0.8 ) |
| Asthenia* | 246 | 2.07 ( 1.82 - 2.34 ) | 2.05 ( 133.73 ) | 2.05 ( 1.85 ) | 1.04 ( -0.63 ) |
| Vomiting* | 212 | 1.48 ( 1.29 - 1.69 ) | 1.47 ( 32.55 ) | 1.47 ( 1.32 ) | 0.56 ( -1.11 ) |
| No adverse event* | 206 | 3.76 ( 3.28 - 4.31 ) | 3.73 ( 411.97 ) | 3.73 ( 3.32 ) | 1.9 ( 0.23 ) |
| Taste disorder* | 197 | 31.27 ( 27.15 - 36.01 ) | 30.97 ( 5637.02 ) | 30.56 ( 27.15 ) | 4.93 ( 3.27 ) |
| Myalgia* | 188 | 3.63 ( 3.14 - 4.19 ) | 3.6 ( 353.87 ) | 3.6 ( 3.19 ) | 1.85 ( 0.18 ) |
| Pain | 177 | 0.82 ( 0.71 - 0.95 ) | 0.82 ( 6.8 ) | 0.82 ( 0.73 ) | -0.28 ( -1.95 ) |
| Headache | 164 | 0.79 ( 0.68 - 0.92 ) | 0.79 ( 8.81 ) | 0.79 ( 0.7 ) | -0.33 ( -2 ) |
| Drug ineffective | 153 | 0.33 ( 0.28 - 0.39 ) | 0.34 ( 203.8 ) | 0.34 ( 0.3 ) | -1.57 ( -3.23 ) |
| Malaise | 141 | 0.92 ( 0.78 - 1.08 ) | 0.92 ( 1.06 ) | 0.92 ( 0.8 ) | -0.12 ( -1.79 ) |
| Rash | 125 | 0.85 ( 0.71 - 1.01 ) | 0.85 ( 3.53 ) | 0.85 ( 0.73 ) | -0.24 ( -1.91 ) |
| Basal cell carcinoma* | 116 | 22.47 ( 18.71 - 27 ) | 22.35 ( 2342.87 ) | 22.14 ( 18.99 ) | 4.47 ( 2.8 ) |
| Dehydration* | 115 | 2.85 ( 2.38 - 3.43 ) | 2.84 ( 137.47 ) | 2.84 ( 2.44 ) | 1.51 ( -0.16 ) |
| Fall | 114 | 1.05 ( 0.87 - 1.26 ) | 1.05 ( 0.27 ) | 1.05 ( 0.9 ) | 0.07 ( -1.6 ) |
| Dizziness | 109 | 0.7 ( 0.58 - 0.84 ) | 0.7 ( 14.46 ) | 0.7 ( 0.6 ) | -0.52 ( -2.19 ) |
| Abdominal pain upper* | 106 | 1.61 ( 1.33 - 1.95 ) | 1.61 ( 24.66 ) | 1.61 ( 1.37 ) | 0.69 ( -0.98 ) |
| Intentional product use issue* | 103 | 3.04 ( 2.51 - 3.69 ) | 3.03 ( 140.46 ) | 3.03 ( 2.58 ) | 1.6 ( -0.07 ) |
| Squamous cell carcinoma* | 102 | 34.4 ( 28.28 - 41.85 ) | 34.23 ( 3241.71 ) | 33.73 ( 28.63 ) | 5.08 ( 3.41 ) |
| Insomnia | 96 | 1.13 ( 0.93 - 1.39 ) | 1.13 ( 1.52 ) | 1.13 ( 0.96 ) | 0.18 ( -1.49 ) |
| Abdominal discomfort* | 89 | 1.53 ( 1.24 - 1.89 ) | 1.53 ( 16.4 ) | 1.53 ( 1.29 ) | 0.61 ( -1.05 ) |
| Pneumonia | 87 | 0.78 ( 0.63 - 0.96 ) | 0.78 ( 5.53 ) | 0.78 ( 0.65 ) | -0.36 ( -2.03 ) |
| Pruritus | 86 | 0.7 ( 0.57 - 0.87 ) | 0.7 ( 10.82 ) | 0.7 ( 0.59 ) | -0.51 ( -2.17 ) |
| Pain in extremity | 81 | 0.81 ( 0.65 - 1.01 ) | 0.81 ( 3.67 ) | 0.81 ( 0.67 ) | -0.31 ( -1.97 ) |
| Dyspnoea | 78 | 0.43 ( 0.34 - 0.53 ) | 0.43 ( 60.1 ) | 0.43 ( 0.36 ) | -1.22 ( -2.89 ) |
| Abdominal pain | 71 | 0.97 ( 0.77 - 1.23 ) | 0.97 ( 0.05 ) | 0.97 ( 0.8 ) | -0.04 ( -1.7 ) |
| Anaemia | 71 | 1.15 ( 0.91 - 1.45 ) | 1.15 ( 1.33 ) | 1.15 ( 0.94 ) | 0.2 ( -1.47 ) |
| Disease progression* | 71 | 1.88 ( 1.49 - 2.37 ) | 1.87 ( 28.97 ) | 1.87 ( 1.54 ) | 0.91 ( -0.76 ) |
| Dysphagia* | 70 | 2.27 ( 1.79 - 2.87 ) | 2.26 ( 49.28 ) | 2.26 ( 1.86 ) | 1.18 ( -0.49 ) |
| Back pain | 70 | 0.91 ( 0.72 - 1.14 ) | 0.91 ( 0.69 ) | 0.91 ( 0.74 ) | -0.14 ( -1.81 ) |
| Gastrointestinal disorder* | 70 | 1.74 ( 1.38 - 2.2 ) | 1.74 ( 21.97 ) | 1.74 ( 1.43 ) | 0.8 ( -0.87 ) |
| Product dose omission issue | 66 | 0.37 ( 0.29 - 0.47 ) | 0.37 ( 72.31 ) | 0.37 ( 0.3 ) | -1.44 ( -3.11 ) |
| Cerebrovascular accident* | 63 | 1.3 ( 1.01 - 1.66 ) | 1.3 ( 4.27 ) | 1.3 ( 1.05 ) | 0.37 ( -1.29 ) |
| Depression | 60 | 0.86 ( 0.67 - 1.11 ) | 0.86 ( 1.32 ) | 0.86 ( 0.7 ) | -0.21 ( -1.88 ) |
| Cough | 59 | 0.63 ( 0.49 - 0.81 ) | 0.63 ( 13.07 ) | 0.63 ( 0.51 ) | -0.67 ( -2.34 ) |
| Pyrexia | 57 | 0.52 ( 0.4 - 0.67 ) | 0.52 ( 25.6 ) | 0.52 ( 0.42 ) | -0.95 ( -2.61 ) |
| Hepatic enzyme increased* | 57 | 2.75 ( 2.12 - 3.56 ) | 2.74 ( 63.11 ) | 2.74 ( 2.2 ) | 1.45 ( -0.21 ) |
| Ill-defined disorder* | 57 | 2.82 ( 2.17 - 3.66 ) | 2.81 ( 66.62 ) | 2.81 ( 2.26 ) | 1.49 ( -0.17 ) |
| Urinary tract infection | 56 | 0.98 ( 0.76 - 1.28 ) | 0.98 ( 0.01 ) | 0.98 ( 0.79 ) | -0.02 ( -1.69 ) |

Abbreviation: Asterisks (*) indicate statistically significant signals in algorithm; ROR, reporting odds ratio; PRR, proportional reporting ratio; EBGM, empirical Bayesian geometric mean; EBGM05, the lower limit of the 95% CI of EBGM; IC, information component; IC025, the lower limit of the 95% CI of the IC; CI, confidence interval; PT, preferred term.
